# EEG Correlates of Long-Distance Dependency Formation in Mandarin *Wh*-Questions

**DOI:** 10.3389/fnhum.2021.591613

**Published:** 2021-02-05

**Authors:** Chia-Wen Lo, Jonathan R. Brennan

**Affiliations:** Department of Linguistics, University of Michigan, Ann Arbor, MI, United States

**Keywords:** Mandarin, long-distance dependency, Wh-question, covert dependencies, EEG, sustained anterior negativity, P600

## Abstract

Event-related potential components are sensitive to the processes underlying how questions are understood. We use so-called “covert” *wh*-questions in Mandarin to probe how such components generalize across different kinds of constructions. This study shows that covert Mandarin *wh*-questions do not elicit anterior negativities associated with memory maintenance, even when such a dependency is unambiguously cued. *N* = 37 native speakers of Mandarin Chinese read Chinese questions and declarative sentences word-by-word during EEG recording. In contrast to prior studies, no sustained anterior negativity (SAN) was observed between the cue word, such as the question-embedding verb “wonder,” and the *in-situ wh*-filler. SANs have been linked with working memory maintenance, suggesting that grammatical features may not impose the same maintenance demands as the content words used in prior work.

## 1. Introduction

A central puzzle in linguistics is how language users understand the relationship between words that appear far apart from each other in sentences. These are called long-distance, or non-local, dependencies. Previous studies have investigated the processing of different kinds of long-distance dependencies, including topicalization (1), relativization (2), and *wh*-questions (3) (e.g., Kluender and Kutas, 1993; Gibson et al., 2005; Phillips et al., 2005). The italicized words in (1)–(3) are so-called “fillers,” which appear in a part of the sentence other than where they are interpreted. The interpretation site, or “gap,” is indicated with underlining. This paper uses *wh*-dependencies, of the sort shown in (3), to probe the processing mechanisms that underlie long-distance dependencies.


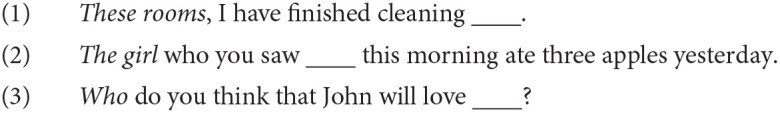


Long-distance dependency formation affects memory in several ways. First, the filler must be maintained in working memory. Indeed, long-distance dependencies show a distance effect: processing time increases as the distance between the filler and the gap increases (Gibson, 2000; Lewis et al., 2006). The maintenance of the *wh*-filler is necessary until the gap site is found and thus working memory capacity is required when processing the filler-gap dependency. Studies have shown that dependencies are recognized actively: comprehenders anticipate a gap as soon as possible after encountering a filler (the “active filler strategy”; Stowe, 1986). Considering the *wh*-question in sentence (3); when the *wh*-word *who* is encountered at the beginning of the sentence, a speaker makes a prediction about the presence of a gap and tries to find the gap as soon as possible.

The above properties reflect processing that is elicited by the filler. Additionally, the filler is retrieved at the appropriate part of the sentence and its thematic role and grammatical function needs to be resolved. Drawing the processing evidence together, long-distance *wh*-dependencies involve the following four elements: (i) *wh*-filler reactivation, (ii) thematic role assignment, (iii) semantic integration between the verb and the *wh*-filler, and (iv) maintenance of a *wh*-filler and prediction of the incoming gap (Kaan et al., 2000; Phillips et al., 2005; Wagers and Phillips, 2014; Omaki et al., 2015). The manner by which these elements are accomplished differs across languages. For example, some languages such as Japanese and German have overt morphological or lexical markers to indicate the thematic role of the *wh*-filler while other languages such English and Mandarin Chinese do not have any overt markers. In electrophysiological research, these different elements have been associated different event-related potentials (ERP) components. The current study focuses on ERP components that have been connected with the maintenance of a filler element in working memory; we provide novel data from the processing of Mandarin *wh*-questions to shed light on the functional interpretation of these components.

### 1.1. Long-Distance Dependencies and the Sustained Anterior Negativity (SAN)

The Sustained Anterior Negativity (SAN) is an ERP component that appears to correlate with storage and retrieval of a *wh*-filler. The SAN component is an increased negative voltage over the anterior of the scalp between the interval of interest (i.e., the interval that a dependency needs to be maintained) post-stimulus and is associated with maintenance of information in working memory (Fiebach et al., 2002; Phillips et al., 2005). Specifically, the SAN has been linked with the maintenance of an incomplete dependency and the length of the dependencies. Fiebach et al. (2002) examined the processing of German *wh*-questions and find a centrally-localized SAN between the cue-word (e.g., *wh*-filler) and the gap. They found that the amplitude of the SAN became larger as the distance from the *wh*-filler became greater, a finding that they explain by suggesting that it is more costly to maintain the incomplete dependency in working memory. The SAN is thus associated with the cost of holding information available in working memory since information stored in working memory declines with time (Fiebach et al., 2002).

Phillips et al. (2005) observed a SAN in both short and long *wh*-dependencies with a greater amplitude of SAN in the long dependencies. The SAN effect is seen between the *wh*-filler and the gap position, consistent with the idea that the SAN is associated with the cost of retaining the *wh*-filler in working memory. Interestingly, their SAN effect was already observed in the first clause of the long *wh*-dependencies. They point out that it is insufficient to conclude that the SAN can be evoked by the manipulation of the length of *wh*-dependencies as the SAN amplitude only increases in the first clause and is sustained, but not further increased subsequently. While not identical, this result is consistent with the results from Fiebach et al. (2002): for both studies a SAN was elicited when a long-distance dependency is cued.

In contrast, some studies have failed to find a SAN effect between a filler and a gap (McKinnon and Osterhout, 1996; Kaan et al., 2000). Kaan et al. (2000) present an experiment using grammatical indirect questions with three conditions: (a) *who*-questions, (b) *whether*-questions, and (c) *which N*-questions; examples are given in (4). For *wh*-fillers such as “who” and “which,” the SAN effect was predicted to be evoked between the filler words and the corresponding gaps since the incomplete dependency must be maintained until the gap is encountered. To explain the absence of such a sustained effect in their data, Kaan and colleagues suggest that expectations of some optional phrases such as “or not” could minimize processing differences between the *wh*-filler conditions and the *whether*-condition.


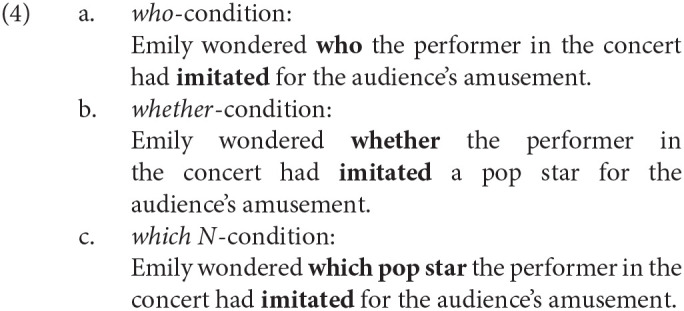


The inconsistency between previous findings about the SAN for English indirect questions render unclear how different kinds of information may modulate working memory maintenance, and thus the elicitation of a SAN. Lau (2018) identifies two possible kinds of information whose maintenance could drive the SAN: (i) the maintenance of the *wh*-feature, or (ii) the maintenance of the syntactic prediction for filler itself. These two hypotheses differ in terms of the type of information that is driving this observation: for the former, the information is born by the *wh*-feature itself. In the latter, the relevant information is born by the linguistic content that is actively maintained to fill the gap site. Crucially, English *wh*-dependencies cannot tease apart these two possibilities. The existing data leave open whether a SAN effect could only be elicited by the maintenance of the *wh*-feature, of a contentful word. The present study examines the processing of Mandarin *wh*-questions, which has the *wh*-feature to trigger the prediction of the *in-situ wh*-word encountered later. In doing so, we can tease apart whether the SAN effect is elicited either by the *wh*-feature only or by the prediction formed by the tail of a dependency.

### 1.2. Long-Distance Dependencies and the P600

A secondary goal of this study touches on another ERP component associated with the processing of *wh*-dependencies: the P600. This component begins about 600 ms after the onset of a target word with a central-parietal scalp distribution (Osterhout and Holcomb, 1992, 1993; Hagoort et al., 1993). The P600 has been associated with several functional roles, including syntactic reanalysis in ungrammatical or less preferred structures (Osterhout and Holcomb, 1992, 1993), thematic analysis, and syntactic integration (Kaan et al., 2000; Phillips et al., 2005). Theoretical frameworks differ in whether they associated the P600 with combinatorial mechanisms (Kuperberg, 2007), or with memory operations, as in the retrieval-integration account of Brouwer et al. (2012, 2017).

In terms specific to *wh*-dependencies, the P600 has been associated with the integration of the filler word with the gap site. Kaan and colleagues propose that the P600 elicited at the gap reflects a process of syntactic integration, by which they mean “the amount of energy used to reactivate previous predictions and integrate them into the current input” (Kaan et al., 2000, p. 163). In the sentences in (4), all conditions require such syntactic integration between the verb and the noun. The embedded verb *imitated* assigns a thematic role agent to the previous NP *the performer in the concert*. However, in (4-a) and (4-c), extra integration is required between the *wh*-element and the embedded verb *imitated* in terms of thematic role assignment to the *wh*-element. The embedded verb needs to assign the thematic role patient to the previous encountered *wh*-word *who*. The results are consistent with a functional role of the P600 that is linked with thematic analysis and/or syntactic integration. Kaan et al. further report a second experiment in which grammatical indirect questions (with *who* and *whether*) were paired with ungrammatical indirect questions that had agreement violations. In addition to the elicitation of the P600 at the embedded verb in the grammatical indirect questions, an P600 was also found at the embedded target verb in the ungrammatical sentences. Taken together, these findings strongly suggest that the P600 is not restricted to unpredicted structure alone, but can be elicited by grammatical and predictable structure that none-the-less imposes a greater burden on syntactic integration.

Building on this, Phillips et al. (2005) suggest a restricted view of integration for the P600. As already mentioned above, they investigated English *wh*-dependencies by manipulating the length between filler and gap. Their EEG results show that the P600 occurs around 300–500 ms in the short *wh*-dependencies but at 500–700 ms in the long *wh*-dependencies, suggesting that the differences in latency might indicate the timing for reactivating the *wh*-filler. However, there is no significant difference in the peak amplitude of the P600 when the dependency is finished in *wh*-dependencies, regardless of the short *wh*-dependencies or long *wh*-dependencies. They interpret this result to suggest that the amplitude of the P600 seems to be associated with the subprocesses of thematic role assignment and/or semantic integration between the verb and the *wh*-filler.

Data from findings discussed thus far come from languages in which the long-distance dependency formed in *wh*-questions is “overt.” This contrasts with languages, like Mandarin, where the *wh*-element stays *in-situ* but is none-the-less found to have similar syntactic properties as overt-movement languages. If the P600 relates to the reactivation of the *wh*-filler, we should not expect to see the P600 in the Mandarin *wh*-questions. Unlike English *wh*-questions, the *wh*-element in Mandarin *wh*-questions stays *in-situ*, as detailed in section 1.3. However, if the P600 relates to the integration cost and thematic role assignment, the predictions are more complicated as they depend on the relative cost of integration between a verb and different kinds of arguments. If the integration cost of a question word is greater than a non-question word, perhaps due to interference effects during retrieval (Xiang et al., 2015), then we would expect the P600 would be elicited in Mandarin *wh*-questions.

### 1.3. Overt and Covert Long-Distance Dependencies

**Figure 1:**
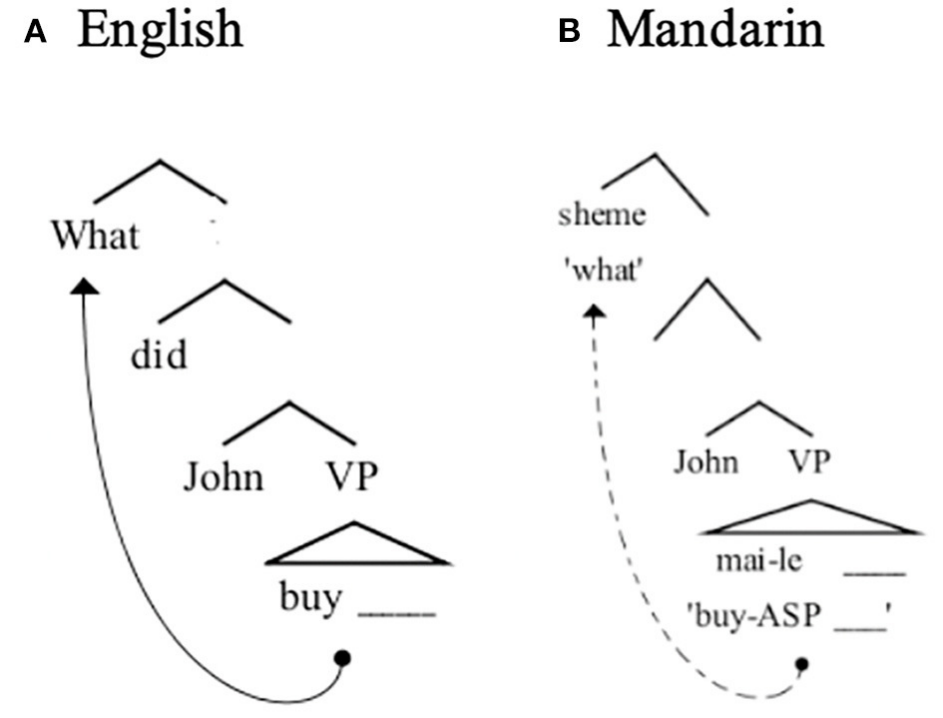
Syntactic representations for **(A)** English *wh*-questions and **(B)** Mandarin *wh*-questions.

To understand how Mandarin *wh*-question helps to meet the goals of the present study, we next briefly discuss some key syntactic and psycholinguistic aspects of Mandarin questions. Mandarin *wh*-questions have a very different word order than equivalent questions in English. However, as shown in Figure 1, this surface difference belies an underlying similarity; the *wh*-elements in English and Mandarin are claimed to be at the same syntactic position relatively high in the clause. Under this view, the only difference between these two languages is that the dependency in Mandarin takes place at the level of semantic interpretation, but not at the level of phonological spell-out (hence the term “covert movement”). (5-a) shows that the English *wh*-element *what* moves from the verb phrase (VP) to a clause-initial position. We review, below, evidence suggesting that the Mandarin *wh*-element *sheme* “what” behaves in a syntactically parallel way to English in that it moves to a clause-initial position despite the fact that such movement is not apparent in the overt word order of the sentence (5-b) (Huang, 1982).


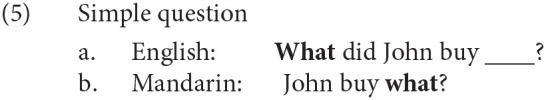


The key evidence that Mandarin *wh*-question involves covert movement comes from the scope-taking properties of *wh*-elements associated with different kinds of verbs. Huang (1982) points out that Chinese, like English, shows similar selectional restrictions between verbs and complements, as illustrated in (6) and (7). In the English examples (6), it is easy to distinguish the direct questions from the indirect questions since they have different surface word orders. However, in Mandarin (7), both the direct question and the indirect question have the same surface word order. If the main verb selects for an interrogative complement (e.g., *xiang-zhidao* “wonder”), the sentence forms an embedded, or “indirect,” question interpretation (Huang, 1982). If the main verb is a non-question verb (e.g., *yiwei* “think”), the sentence forms a direct question. Huang argues that this interpretive difference follows from a structural difference. Huang suggests that if the *wh*-element moves (covertly) to front of the embedded clause, the sentence is interpreted as an indirect question. However, if the *wh*-element moves covertly to the front of the main clause, it forms a direct question. This hypothesis has theoretical parsimony: the different interpretation between the direct question and the indirect question is achieved in the same way in English and Mandarin. Therefore, Huang concludes that the *wh*-element moves but as this movement is reflected only in interpretation and not in surface word order, hence “covert.”


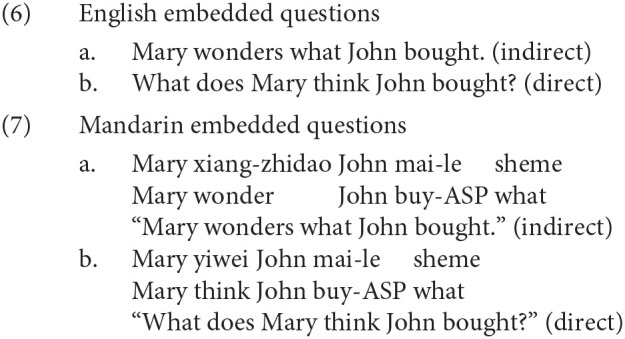


Indeed, previous findings have shown that memory resources required by questions are similar between the two languages (Xiang et al., 2014, 2015). Xiang et al. (2014) used the speed-accuracy tradeoff method, which allows experimenters tease apart response accuracy and response speed by polling for responses over time. The results reveal that long *wh*-questions show lower asymptotic accuracy than the short *wh*-questions. That is, relative to the short *wh*-questions, the latency of response time is delayed for the long *wh*-questions when the asymptotic accuracies are equal. The observation Mandarin *wh*-questions show such a distance effect is consistent with the hypothesis that the parser needs to construct a covert dependency.

Moreover, Xiang et al. (2015) used a similarity-based interference paradigm to probe the memory consequences of material that falls within the span of the covert dependency (see Van Dyke and Lewis, 2003). They found that intervening material that was similar to the *wh*-filler but appeared between the question-selecting verb (e.g., *wonder*) and the *wh*-word increased processing load in a way similar to what has been previously observed for English overt long-distance dependencies (McElree et al., 2003). Specifically, their eye-tracking and self-paced reading results revealed that the intermediate embedded clause in multiple-embedding conditions can interfere with the dependency between the questions-selecting verb and the *wh*-element in the lowest clause. The findings suggest that covert *wh*-dependencies indeed require maintenance of a dependency, and that covert *wh*-movement leads to an increased processing load in a way similar to overt long-distance dependencies in English. In other words, memory retrieval for a covert dependency resembles that of an overt dependency since both are subject to interference effects. The difference is that the retrieval process of an overt dependency is led by overt morpho-syntactic cues while the retrieval process of a covert dependency is led by structural cues.

The above findings indicate that formation of covert *wh*-dependencies can be detected in on-line sentence processing. One prior study has used ERPs to examine covert dependency processing using the *wh*-*in-situ* language Japanese (Ueno and Kluender, 2009). Ueno and Kluender find a sustained anterior negativity that occurs in the right hemisphere between the *wh*-filler and the question particle in *wh*-questions, compared to the yes/no questions in Japanese (they dubbed as “RAN”). They argue that the RAN effects they found should be comparable to SAN effects discussed in section 1.1. They speculate that this hemispheric bias may be due to the non-alphabetic Japanese kanji writing system. Interestingly, Ueno and Kluender do not find a P600 at the *wh*-word, in contrast to prior work with English (discussed in section 1.2). This absence suggests that the P600 might be elicited due to the overt movement of the *wh*-filler: since the *wh*-word stays *in-situ* in Japanese *wh*-questions, they claimed that there is nothing to be integrated at the clause-final question particle and thus no P600 is elicited in the Japanese *wh*-questions.

### 1.4. Predictions for the Present Study

**Figure 2:**
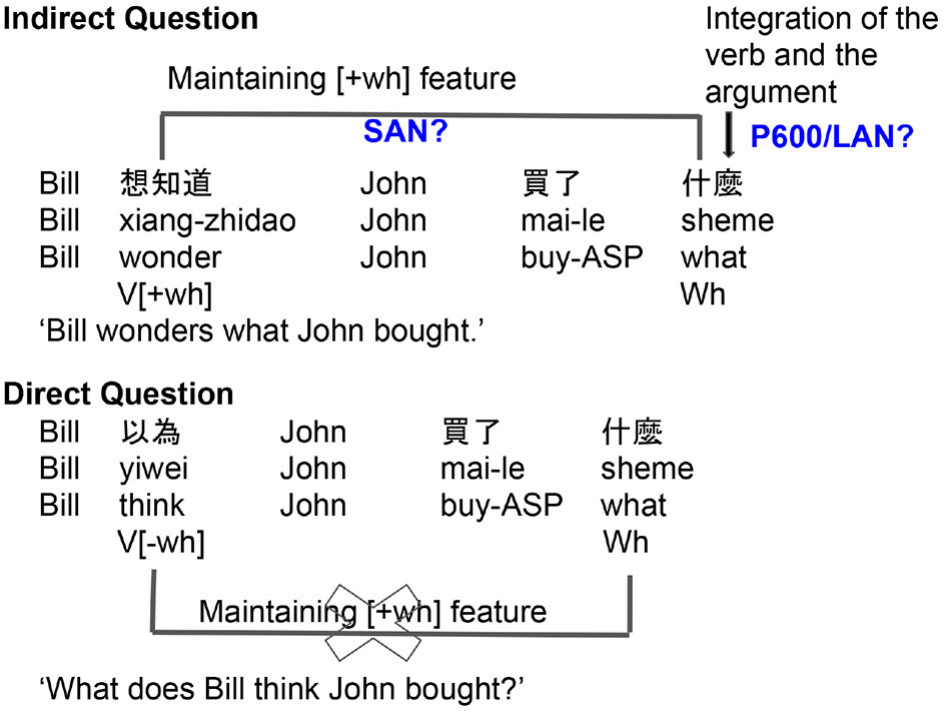
Predictions for ERP correlates of Mandarin *wh*-dependencies with question-embedding or non-question embedding matrix verbs.

**Figure 3:**
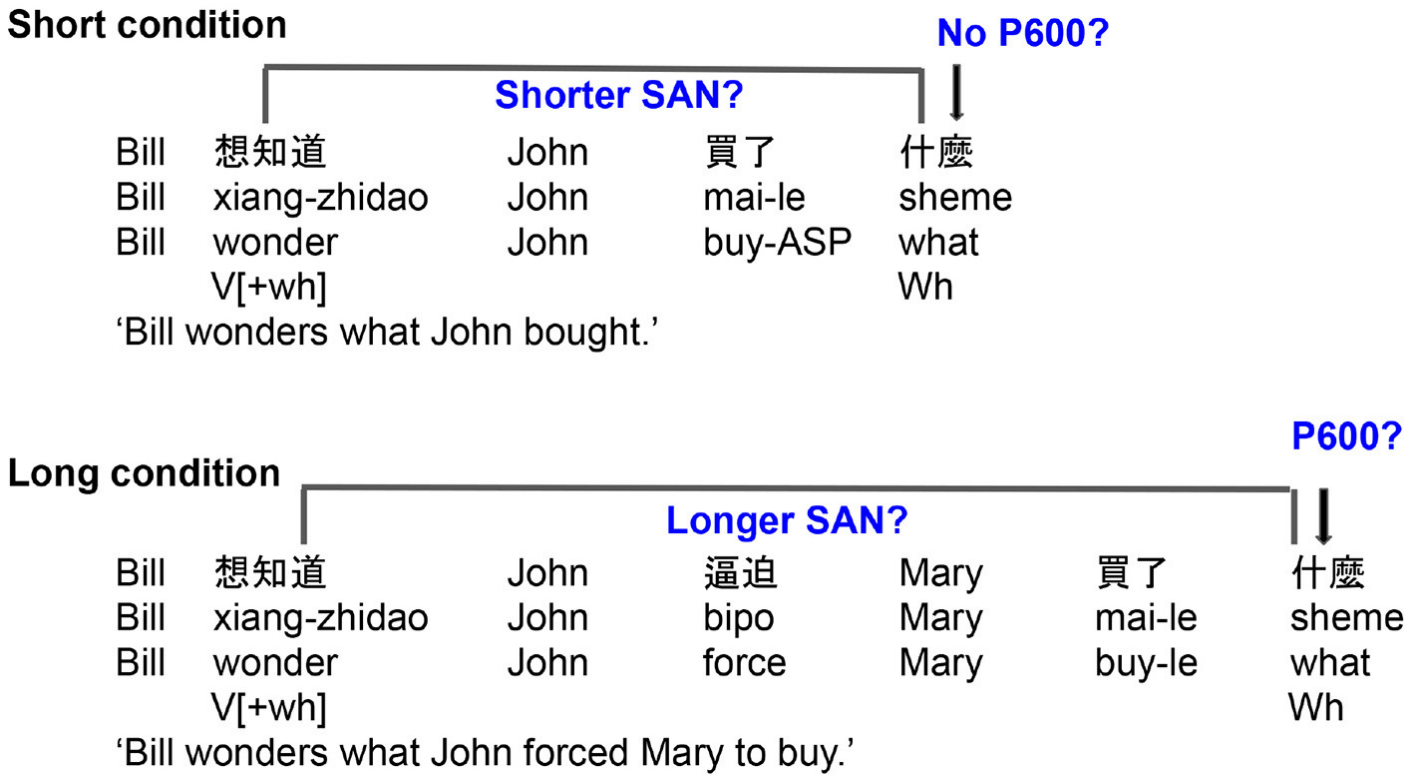
Predictions for ERP correlates of Mandarin *wh*-dependencies as a function of the length of the embedded clause.

The present study tests how Mandarin speakers process *wh*-questions by examining indirect questions, direct questions, declarative sentences, and whether-questions, with two different length of *wh*-dependencies. The background literature we have reviewed offers concrete predictions for the SAN ERP component in Mandarin *wh*-*in-situ* questions. If the parser takes a question-selecting verb as a cue for an upcoming covert dependency, a SAN effect should be elicited between that verb and the embedded *wh*-element in the indirect questions, relative to direct questions or declarative control sentences (see Figure 2). However, if the feature from a question-selecting verb, unlike the storage of the contentful word in English and also Japanese *wh*-questions, requires less (or no) working memory demands, a SAN effect is not expected to appear. In other words, if the SAN effect can be elicited in the indirect questions, this can suggest that the SAN effect can be elicited for maintaining the *wh*-dependency itself, not a particular contentful memory object. In terms of length effects, a greater SAN effect is expected to be observed in the comparison of indirect questions and direct questions in the long condition, compared to the comparison of indirect questions and direct question in the short condition (see Figure 3).

We also consider the role of the P600 in Mandarin *wh*-questions, although the theoretical stakes are not as clear cut. The P600 has been associated with three processing elements: (i) the reactivation of the *wh*-filler, (ii) thematic role assignment, and (iii) semantic integration between the verb and the *wh*-filler, regarding the processing of the overt *wh*-dependencies. Under the reactivation hypothesis, like Japanese *wh*-questions, the P600 should not be elicited since there is no overt displacement for the *wh*-filler in Mandarin. However, the semantic integration and thematic role assignment hypotheses, together with the results of Xiang et al. (2014) and Xiang et al. (2015) showing that question-selecting verb such as “wonder” forms a dependency with the *in-situ wh*-word, predict that a P600 should be found at the *wh*-word in such indirect questions, compared to the direct questions and the declarative sentences (see Figure 2).

## 2. Experiment

### 2.1. Participants

Forty-three native speakers (23 females, 20 males) of Mandarin Chinese between the age of 21 and 40 (*M* = 27) participated in the study at the University of Michigan. They were all right-handed and had normal or corrected-to-normal vision. They self-reported that they did not have any neurological disorders. They gave informed consent and were reimbursed for their experiment time (15 USD/hour). Data from five participants was excluded from the analysis because of low accuracy on the comprehension questions (<80%). Data from one participant was excluded due to having many problematic noisy trials (>20%). Thus, data from 37 participants (18 females, 19 males) were included in the final analysis. The experiment was conducted in accordance with the University of Michigan Health Sciences and Behavioral Sciences Institutional Review Board (# HUM00081060).

### 2.2. Materials

Experimental sentences were created by manipulating two factors. The first factor is whether the main verb is a question-selecting verb (e.g., *wonder*) or a non-question verb (e.g., *think*). If the main verb is a question-selecting verb, the whole sentence forms an indirect question. Otherwise, the sentence forms a direct question. The main verbs were matched on the number of strokes of a word and the log frequency of the first and the second character from SUBTLEX-CH (Cai and Brysbaert, 2010, see Appendix 1). A significant difference [*t*_(21.7)_ = 4.59, *p* < 0.001] was found in the log word frequency between question-selecting verbs (M = 1.8, SD = 0.67) and non-question verb (M = 3.1, SD = 0.82) since we wanted to make sure that the question-selecting verbs introduce a *wh*-feature afterwards and thus the question-type verbs we selected all included the character *wen* “ask.” This type of verb is not as common as the non-question verbs. For this type of verb, it is possible to can take several different types of complements. Based on Xiang et al. (2015), they report data from a series of sentence completion tasks showing that Mandarin speakers are most likely to complete the complement of a wonder-type verb with a *wh*-question. A table showing how variables were matched is given in Appendix 2. The second factor is the length of the sentence: short or long. Compared to the short version of the experimental sentences, the long version of the sentences includes an extra embedded clause. Adapted from Xiang et al. (2015), the verbs in the embedded clauses were transitive verbs which do not select an interrogative clausal complement. As such, these verbs do not intervene between the dependency of the main verb and the *wh*-word. The embedded verbs were selected from the Sinica Corpus (CKIP, 1995) using the tagging VE (i.e., transitive verbs) or VF (i.e., transitive verb or ditransitive verb).

An example of the experimental sentences is given in Table 1, rows (a)–(d). In addition to the two target experimental conditions, declarative sentences and sentences with an embedded yes/no question were included as control conditions [Table 1, rows (e)–(h)]. The declarative sentences (e–f) use the same main verb as the direct questions (e.g., *diaocha* “investigate”). But, the embedded clause does not have any question words, thus there is no long-distance dependency. This serves as a control for the target direct questions. The embedded yes/no questions (g-h) combine the same verbs from the indirect questions (e.g., *xunwen* “ask”) with the question word *you-mei-you* “whether” to serve as a control for the wonder-type sentences. In both Indirect Question and Whether condition, the complement of the matrix verb is a CP; and they are differentiated by the presence of a *wh*-word, or a functional item like *you-mei-you*, respectively.

**Table 1:** Examples of the experimental sentences.

(a) **Short indirect question:** Zhangwei xunwen qianjitian Yongching kai-ASP naxie xianli Zhangwei ask few-days-agoYongching create which precedent “Zhangwei asked which precedents Yongching created a few days ago.”
(b) **Long indirect question:** Zhangwei xunwen qianjitian Yongching bipuo Chinghua kai-ASP naxie xianli Zhangwei ask few-days-ago Yongching force Chinghua create which precedent “Zhangwei asked which precedents that Yongching forced Chinghua to create a few days ago.”
(c) **Short direct question:** Zhangwei diaocha qianjitian Yongching kai-ASP naxie xianli Zhangwei investigate few-days-ago Yongching create which precedent “Which precedents did Zhangwei investigate Yongching create a few days ago?”
(d) **Long direct question:** Zhangwei diaocha qianjitian Yongching bipuo Chinghua kai-ASP naxie xianli Zhangwei investigate few-days-ago Yongching force Chinghua create which precedent “Which precedents did Zhangwei investigate Yongching force Chinghua to create a few days ago?”
(e) **Short declarative:** Zhangwei diaocha qianjitian Yongching kai-ASP zhexie xianli Zhangwei investigate few-days-ago Yongching create these precedent “Zhangwei investigated Yongching created these precedents a few days ago.”
(f) **Long declarative:** Zhangwei diaocha qianjitian Yongching bipuo Chinghua kai-ASP zhexie xianli Zhangwei investigate few-days-ago Yongching force Chinghua create these precedent “Zhangwei investigated Yongching forced Chinghua to create these precedents a few days ago.”
(g) **Short embedded yes/no question:** Zhangwei xunern qianjitian Yongching you-mei-you kai-ASP xianli Zhangwei ask few-days-ago Yongching have-not-have create precedent “Zhangwei asked whether Yongching created precedents a few days ago.”
(h) **Long embedded yes/no question:** Zhangwei xunwen qianjitian Yongching you-mei-you bipuo Chinghua kai-ASP xianli Zhangwei ask few-days-ago Yongching have-not-have force Chinghua create precedent “Zhangwei asked whether Yongching forced Chinghua to create precedents a few days ago.”

One hundred and twenty sentence sets were created initially and organized into eight lists. These were submitted to an acceptability judgment norming study. Twenty-four native Mandarin speakers recruited from social media (6 males and 18 females) judged the sentences on-line through Qualtrics (Qualtrics, Provo, UT). Data were collected anonymously. Subjects were instructed to rate the sentences from 1 (least acceptable) to 5 (most acceptable). Forty ungrammatical filler sentences were also included in this norming test. The stimuli were presented randomly and fully counterbalanced such that each participant saw a list including 120 target sentences and 40 fillers. The norming test took 20–30 min to finish.

Sixty-four acceptable sentence sets were selected based on the results (Overall M = 3.83, SD = 0.74). While these sentence sets were judged to be acceptable overall, some conditions were judged to be more acceptable than others. A two-way analysis of variance shows a main effect for length, *F*_(1, 504)_ = 63.51, *p* < 0.001, such that the average acceptability rating was higher for the short version (*M* = 4.08, SD = 0.68) compared to long version (*M* = 3.59, SD = 0.71). The main effect of sentence type also shows significance, *F*_(3, 504)_ = 3.6, *p* < 0.05. A *post-hoc* Tukey's test shows that the direct question sentences are significantly different from the other three sentence types (adjusted *p* < 0.05), such that the direct question (M = 3.68, SD = 0.75) is less acceptable than the indirect question (*M* = 3.95, SD = 0.69). The declarative (*M* = 3.82, SD = 0.78) and the yes/no question (*M* = 3.88, SD = 0.71) are not different from the indirect question. Note that the differences between indirect question and direct question work against the prediction that indirect question will be costly to process due to increased working memory maintenance cues by the question-selecting verb.

Eight lists were created out of these 64 sentence sets. Sentences were distributed across these lists in the following way, which balances two constraints: ensuring participants see sufficient trials per condition, and minimizing the overall length of the experiment. First, each condition was divided into eight chunks of eight sentences each. Each list contains six chunks, or 48 sentences, from each condition (six sentences from each set), for a total of 384 experimental sentences. The chunks were distributed across the lists according to a Latin square. One hundred and twenty grammatical fillers were also included (80 sentences including a serial verb construction and 40 sentences including a relative clause). Therefore, each participant saw 504 sentences in total.

### 2.3. Procedure

Participants sat comfortably in front of a computer screen in a quiet room. Prior to the main session, participants were fitted with an electrode cap. Electrodes were also affixed above and below the left eye. Electrolyte gel was applied to minimize impedance. The set-up process took about 30 min.

Before the main experiment, participants chose if they would like to read a version using traditional or simplified Chinese characters. Participants were instructed to read the sentences appearing word by word on the computer screen. Sentences were presented with PsychoPy2 (v1.84.2; Peirce, 2007, 2009). Participants were instructed not to move their body or blink their eyes when the stimuli were presented. For each sentence, a 500 ms fixation was presented before the onset of the first word. Every word was presented for 300 ms with a 300 ms inter-stimulus-interval. After the trial, a yes-no comprehension question related to the sentence appeared with a one in eight chance. Participants had the option of a short break after every 10 sentences. After the instructions, participants had a practice session to make sure that they were familiar with the whole procedure of the experiment. During the main session, the experimenter paused the recording at least once to check electrode impedance. The whole recording session took about an hour. After the main session, participants washed their hair to remove electrolyte gel and were given debriefing about the goals of the experiment.

### 2.4. EEG Recording and Processing

EEG was recording from 61 active electrodes at 500 Hz (actiCHamp, BrainProducts GmbH) in a 0.1–200 Hz band with on-line reference to an electrode placed on the right mastoid. Impedance were kept below 25 kOhms. FieldTrip software (Oostenveld et al., 2011) was used to analyze the data. Offline, the data were divided into epochs around word onset and were re-referenced to the average of the left and the right mastoid electrodes. Artifacts related to eye blinks were removed via Independent Component Analysis (Makeig et al., 1995; Jung et al., 2000) and remaining artifacts were removed manually following visual inspection. Signals from electrodes with supra-threshold impedance or exceptional noise were replaced by using surface spline interpolation (Perrin et al., 1989).

Prior work studying the SAN and P600 components (e.g., Ueno and Kluender, 2009) set a bandpass filter from 0.2 to 15 Hz. However, a 0.2 lower-cutoff might attenuate differences for a slow wave, such as a SAN that spans multiple words. This study applied a low-pass filter at 20 Hz to the epoched data.

### 2.5. Statistical Analysis

A non-parametric permutation test (Maris and Oostenveld, 2007) was conducted across all electrodes time-locked to (i) the target *wh*-word, or (ii) the interval between the main verb and the *wh*-word to examine the dependency between the main verb and the *wh*-filler. The epoch around the target *wh*-word included a 0.3 s pre-stimulus baseline and a 1 s post-stimulus period. The non-parametric permutation test was used to correct for multiple comparisons across timepoints from 0 to 1 s and across different electrodes by following these steps: (i) One-way repeated measures ANOVAs were conducted at each timepoint and electrode, (ii) tests with *p* < 0.05 were clustered based on spatial-temporal adjacency and their F-statistics were summed, (iii) Steps (i) and (ii) were repeated 1,000 times by randomly permuting the condition labels for each subject, and (iv) clusters with summed statistics that surpassed at least 95% from the permutation test were kept as “statistically significant.” Intervals between the verb and the *wh*-word start from 0.3 s prior to the main verb [e.g., “ask” in Table 1 (a)] and spans 2.2 s, which extends across the presentation of the three words following the main verb [e.g., “few-days-ago Yongching create” in row (a) of Table 1].

For each comparison, a first analysis involved a one-way ANOVA comparing Indirect questions, Direct questions, and Declarative sentences, averaging across the Long and Short versions of each item. Whether-questions are excluded in this comparison to ensure a close match between target word regions. The first goal of this study is to examine whether a sustained anterior negativity is found in *wh*-dependencies that are cued by the question-selecting verb in Indirect questions, compared to Direct questions and Declarative sentences where there is no such cue. We additionally test for whether a P600 is observed at the *wh*-word in Indirect questions, compared to Direct questions and Declarative sentences. Thus, the length manipulation is left out of consideration for this first goal.

A Bayes factor analysis was also conducted with the BayesFactor package version 0.9.12.4.2 in R (Morey and Rouder, 2018) using default priors. For the SAN, we averaged the ERP amplitudes across the 2,200 ms interval between the onset of the main verb and the *wh*-word/demonstrative in anterior sensors and submitted these to a Bayesian ANOVA. In addition, we averaged the ERP amplitudes across 300–500 ms in anterior sensors for each word between main verb and the *wh*-word. For the P600, the average ERP amplitudes over posterior sensors at 500–700 ms after the *wh*-word/demonstrative were compared.

To test for effects of length, a non-parametric permutation test was conducted across all electrodes time-locked to (i) the target *wh*-word, or (ii) the interval between the main verb and the *wh*-word in short and long conditions separately (see Fiebach et al., 2002; Phillips et al., 2005). Intervals around the target *wh*-word included a 300 ms pre-stimulus baseline and a 1,000 ms post-stimulus interval. Intervals between the verb and the *wh*-word start from 300 ms prior to the main verb (e.g., “ask” in row (a) of Table 1) and goes 2,200 ms, which extends across the presentation of the three words following the main verb for the short conditions (e.g., “few-days-ago Yongching create” in row (a) of Table 1). As for the long conditions, intervals between the verb and the *wh*-word start from 300 ms prior to the main verb and goes 3,000 ms, which extends across the presentation of the five words following the main verb (e.g., “few-days-ago Yongching force Chinghua create” in row (b) of Table 1). For each comparison, the present analysis involved a *t*-test comparing Indirect questions and Direct questions for the Long and Short conditions separately. A Bayes factor analysis was also conducted for the effects of length by following the above time-windows and sensors of interest.

In addition to these evoked analyses, previous research suggests that theta oscillations (4–8 Hz) may reflect the process of retrieval and alpha oscillations (7–13 Hz) can be modulated by the process of storage in working memory in the long-dependency sentences (Meyer et al., 2013, 2015). While our principle focus is on the functional role of evoked components, we also conducted time-frequency analysis to examine oscillations in the theta and the alpha ranges. While the functionality of these frequency bands remain under debate, the analysis serves as a exploratory process to see whether there is a retrieval cost in processing Mandarin *wh*-questions. Details for these analyses are given in the Supplementary Materials.

For visualization, electrodes were divided into six region (numbers are given using the ActiCAP Equidistant 61-channel layout): left anterior (5, 6, 17, 18, 19, 20, 21, 42, 43, 44, 45), anterior midline (1, 7, 33, 34), right anterior (2, 3, 8, 9, 10, 11, 35, 36, 37), left posterior (15, 16, 17, 28, 41, 42, 56, 55), posterior midline (4, 40, 27, 54), and right posterior (12, 13, 14, 26, 38, 39, 52, 53).

## 3. Results

### 3.1. Comprehension Question Accuracy

**Figure 4:**
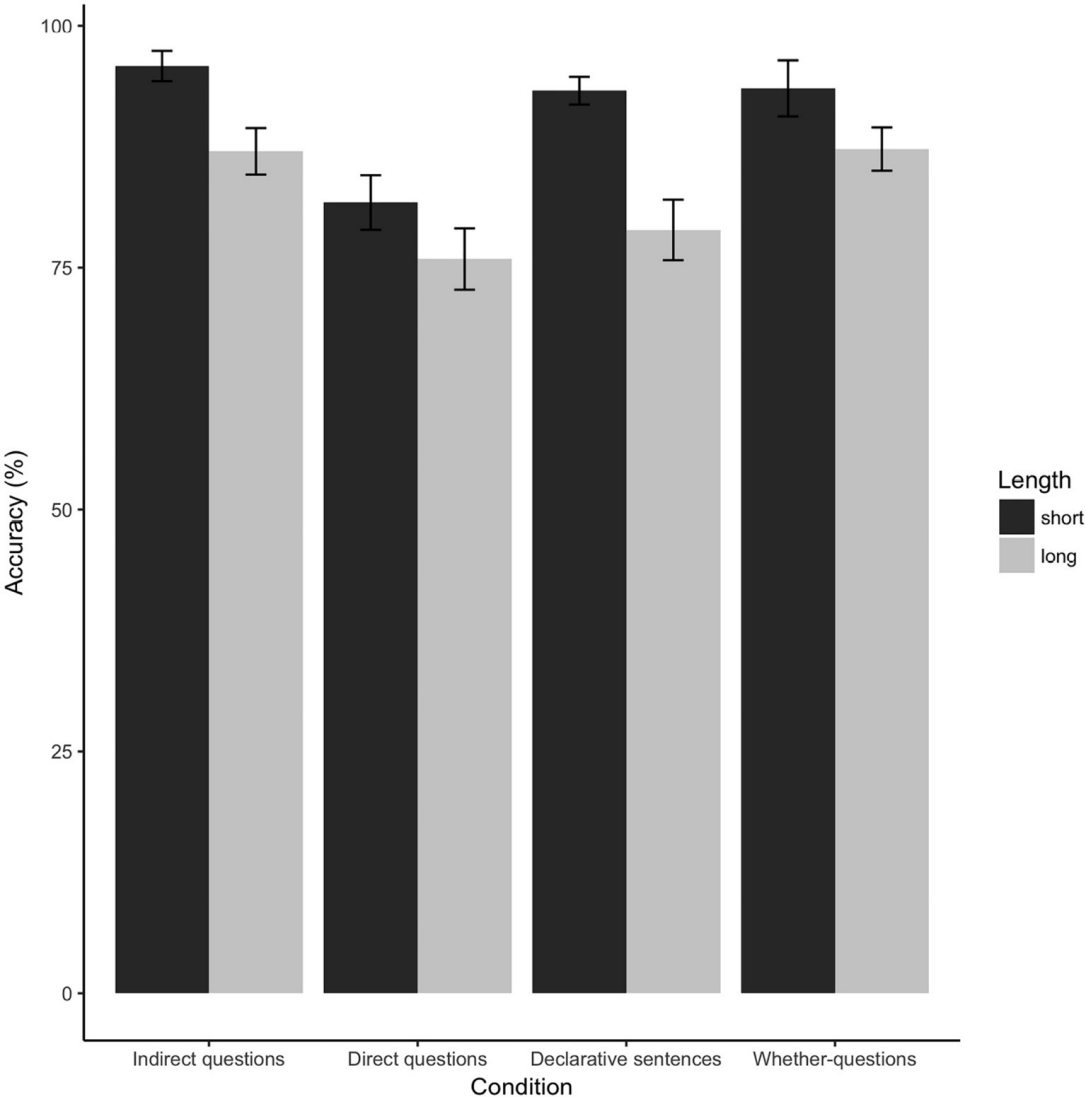
Comprehension question accuracy.

Among 37 participants included in the analyses, the overall accuracy for the target sentences was 89%. Figure 4 shows the accuracy across eight conditions. Overall, the accuracy of short conditions is higher than the accuracy of long conditions.

### 3.2. *Wh*-Dependency—Interval ERP Analyses

**Figure 5:**
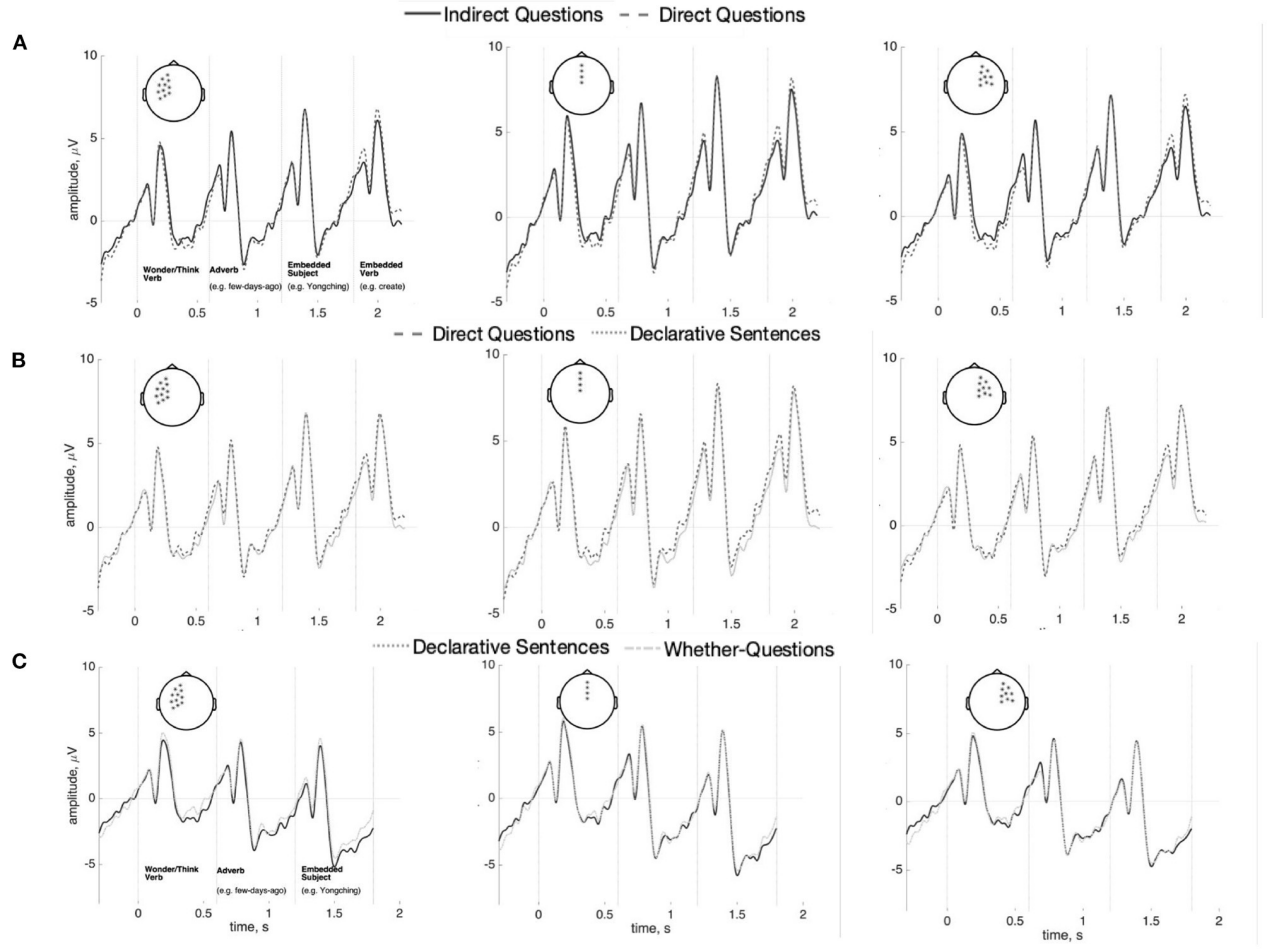
ERPs (n = 37) for the interval between the main verb and the *wh*-word/demonstrative in Indirect Questions vs. Direct questions **(A)**, Direct questions vs. Declarative Sentences **(B)**, and the overlapping regions (i.e., Adverb and Embedded subject) in Indirect Questions vs. Whether-Questions **(C)** at anterior sensors (100 ms baseline computed at the beginning of the interval). There are no statistically reliable effects.

Indirect Questions, Direct Questions, and Declaratives were compared across the interval between the main verb and the *wh*-word to test for a SAN effect. No reliable differences were found between any conditions across this entire interval [min(*p*) = 0.18]. This is shown in Figure 5A (Indirect Questions vs. Direct Questions) and Figure 5B (Direct Question vs. Declarative Sentences). The Bayes factor in favor of the null model is 91.72. This value is generally considered “strong evidence” (Kass and Raftery, 1995). The results of time-frequency analysis also did not reach significance (*p* = 0.87). The figure is shown in Supplementary Figure 1.

A separate comparison compared Indirect Questions with the control whether-question condition (both conditions use the same question-selecting main verb). If the memory maintenance cost for question features does elicit a SAN, such an anterior negativity should be elicited between the question-selecting verb and the words with question-type feature. The interval overlapping in both Indirect questions and Whether-question (i.e., the adverb and the embedded subject) was compared. However, as our results show in Figure 5C, there is no significant difference between the indirect questions and the whether-questions on these words (*p* = 0.67). The Bayes factor in favor of the null model is 1.28 in favor of the null model (“inconclusive” after Kass and Raftery, 1995).

**Figure 6:**
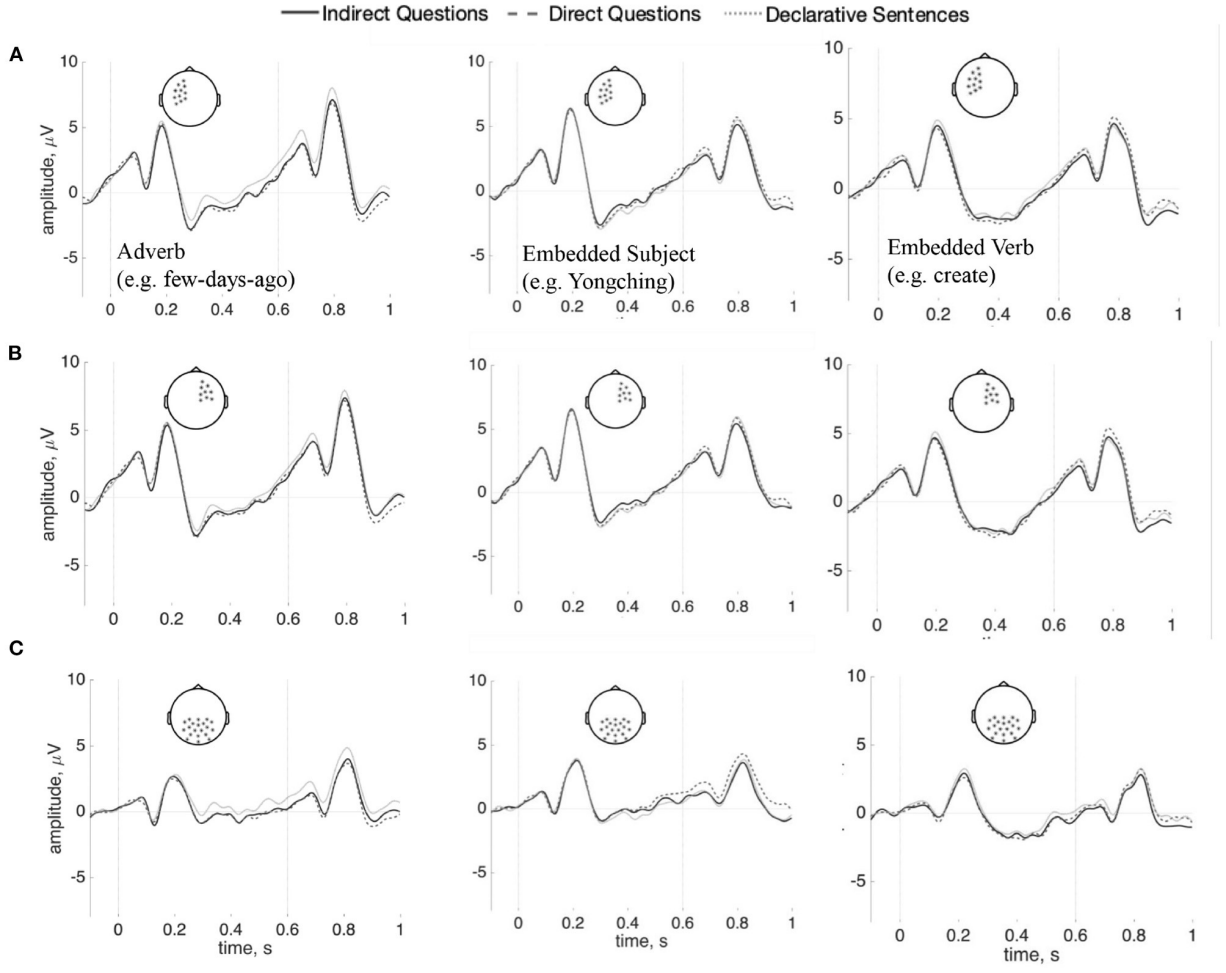
ERPs (*n* = 37) for the adverb (left), the embedded subject (center), and the embedded verb (right) comparing indirect questions, direct questions, and the declarative sentences. **(A)** Shows left anterior sensors; **(B)** shows right-anterior sensors, and **(C)** shows posterior sensors (100 ms baseline computed before each word). There are no statistically reliable effects.

Following Phillips et al. (2005), each word between the main verb and the *wh*-word was also analyzed separately: data epochs were re-baselined with 100 ms before the onset of each word. No reliable differences were found in this analysis (Adverb: *p* = 0.21; Embedded subject: *p* = 0.82; Embedded verb: *p* = 0.54). These analyses show no anterior negativity within the dependency interval, as seen in Figure 6. Bayes factors in favor of the null model for the adverb, embedded subject, and embedded verb regions are, respectively 0.01, 1.39, and 2.57; these data are thus inconclusive regarding differences in the embedded subject and verb regions, while they favor rejecting the null hypothesis in the adverb region. As this latter finding is at odds with the cluster-based test reported above, we believe any evidence for a difference in this one region should be treated with caution. The results of time-frequency analysis also did not reach significance for each word (Adverb: *p* = 0.39; Embedded subject: *p* = 0.78; Embedded verb: *p* = 0.82). The figures are shown in Supplementary Figures 2–4.

To summarize, no SAN was found in the interval between the question-selection verb and the *wh*-element in Indirect questions, compared to Direct questions and Declarative sentences. Also, no anterior negativity was found at each word between the question-selecting verb and the *wh*-element in the Indirect Questions, relative to the Direct questions and the Declarative sentences.

### 3.3. *Wh*-Dependency—Target *wh*-Word ERP Analyses

**Figure 7:**
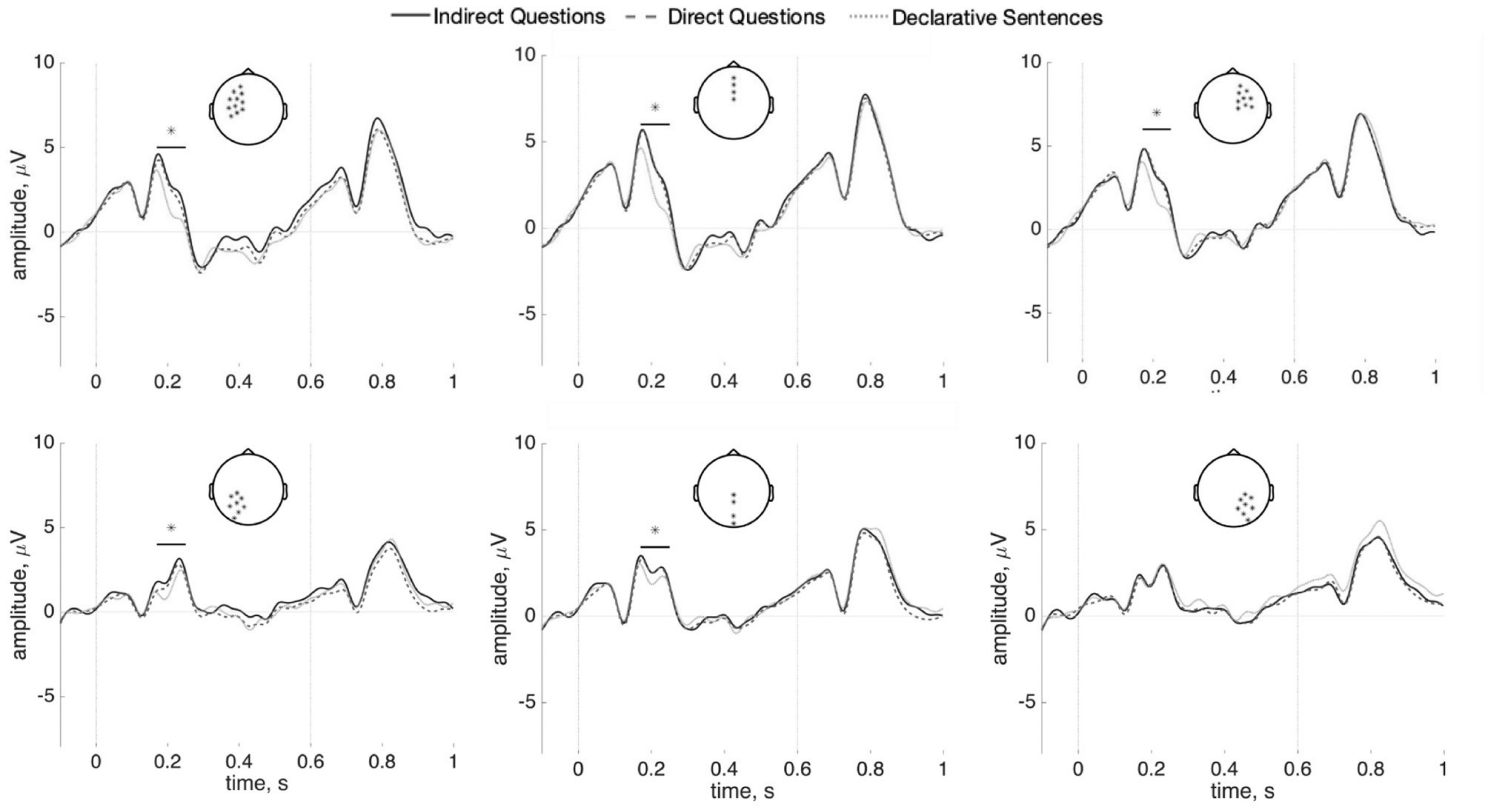
ERPs (*n* = 37) for the *wh*-word *naxie* “which” in the indirect questions/direct questions and the demonstrative *zhexie* “these” in the declarative sentences. Results are shown in left, center, and right channels at anterior **(top)** and posterior **(bottom)** sites. (100 ms prestimulus baseline). Asterisks (*) indicates statistically significant differences.

To test for P600 effects, a one-way repeated-measures ANOVA was computed for the target word region, comparing Indirect questions, Direct questions, and Declaratives. That is, activity evoked by the *wh*-word *naxie* “which” appeared in both the direct question and the indirect question and the demonstrative *zhexie* “these” in the declarative sentences were compared. A significant difference (*p* = 0.03) was found at about 170–250 ms in anterior and left posterior regions. The *wh*-word *naxie* “which” in the indirect questions and the direct questions is more positive-going than the demonstrative *zhexie* “these” in the declarative sentences (see Figure 7). However, there was no P600 nor any anterior negativity at the *wh*-word (*p* = 0.25), relative to the demonstrative *zhexie* “these” in the declarative sentences. The Bayes factor is 0.01 in favor of the difference between conditions and is 89.33 in favor of the null model in the time-window of the P600. The results of time-frequency analysis did not find statistically significance (*p* = 0.44). The figure is shown in Supplementary Figure 5.

### 3.4. Length Effects—Interval ERP Analyses

**Figure 8:**
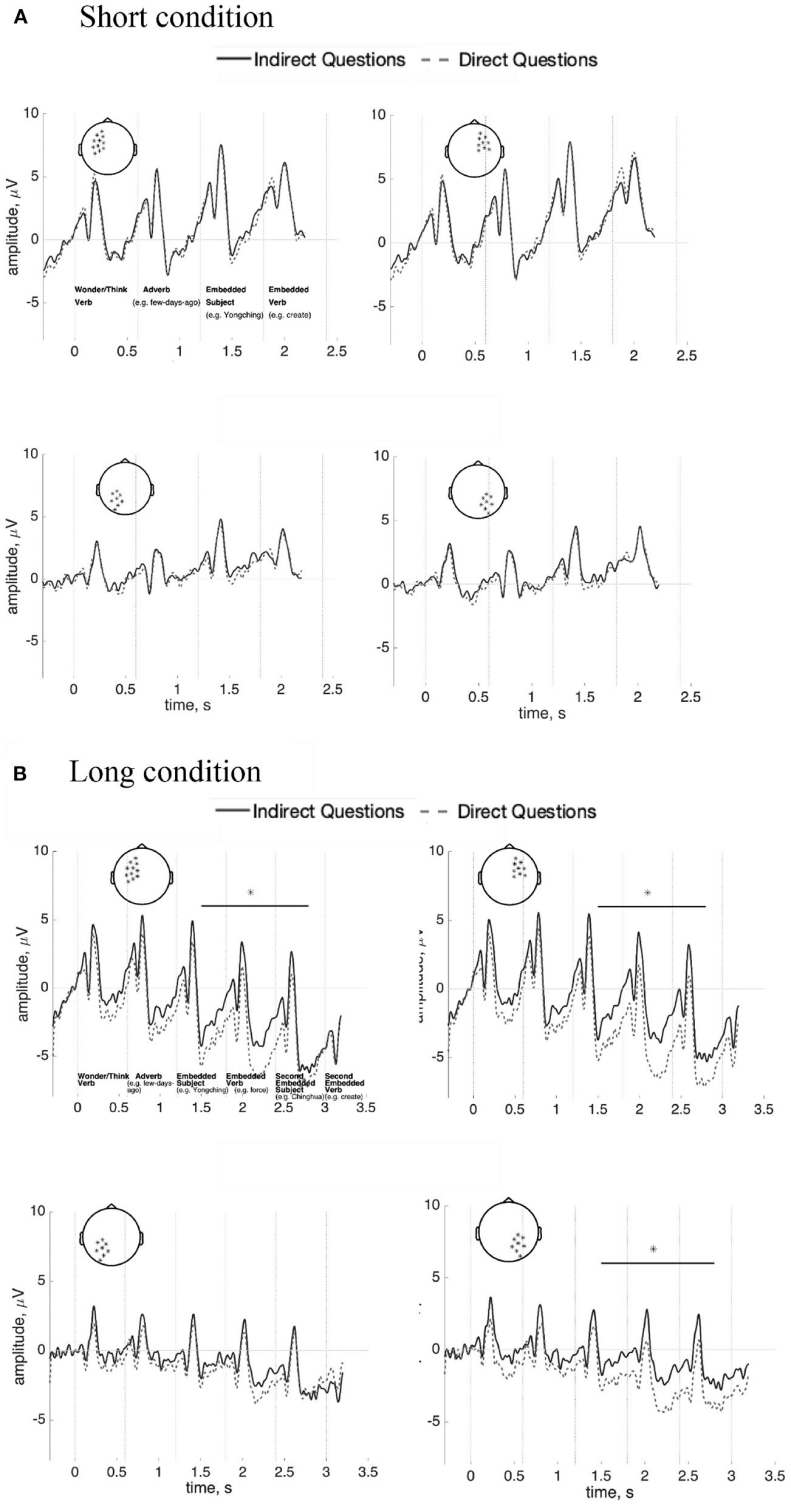
ERPs (*n* = 37) for the interval between the main verb and the *wh*-word *naxie* “which” in the indirect questions and the direct questions across left/right and anterior/posterior sites for the short **(A)** and long **(B)** conditions (100 ms prestimulus baseline). Asterisks (*) indicate statistically significant differences.

The interval between the main verb and the *wh*-word in Indirect question vs. Direct question was also compared in the short (Figure 8A) and long (Figure 8B) conditions. There are no reliable differences between Indirect and Direct questions in the short condition (*p* = 0.67). The Bayes factor in favor of the null model is 9.74 (“substantial evidence”). However, a significant difference was found in the long condition (*p* = 0.03) at approximately 300 ms after the onset of the first embedded subject (i.e., about 1,500–2,800 ms). The effect was found in bilateral anterior and right posterior regions. Figure 8B shows that activity in that interval in Direct questions is more negative than in Indirect questions The direction of this effect contradicts our predictions concerning increased memory maintenance SAN effects cued by the Indirect question-selecting verb. The results of time-frequency analysis show no statistical differences in both short (*p* = 0.72) and long comparisons (*p* = 0.59). The figures are shown in Supplementary Figures 6, 7.

To sum up, no SAN was found in the short indirect questions, compared to the short direct questions, and in long questions a bilateral and posterior sustained positivity was found in approximately 1,500–2,800 ms in the indirect relative to the direct questions. One possible, albeit *post-hoc*, explanation for this sustained positivity might be due to the inhibition of expectation for the *wh*-element. This is further addressed in section 4, below.

### 3.5. Length Effects—Target *wh*-Word ERP Analyses

**Figure 9:**
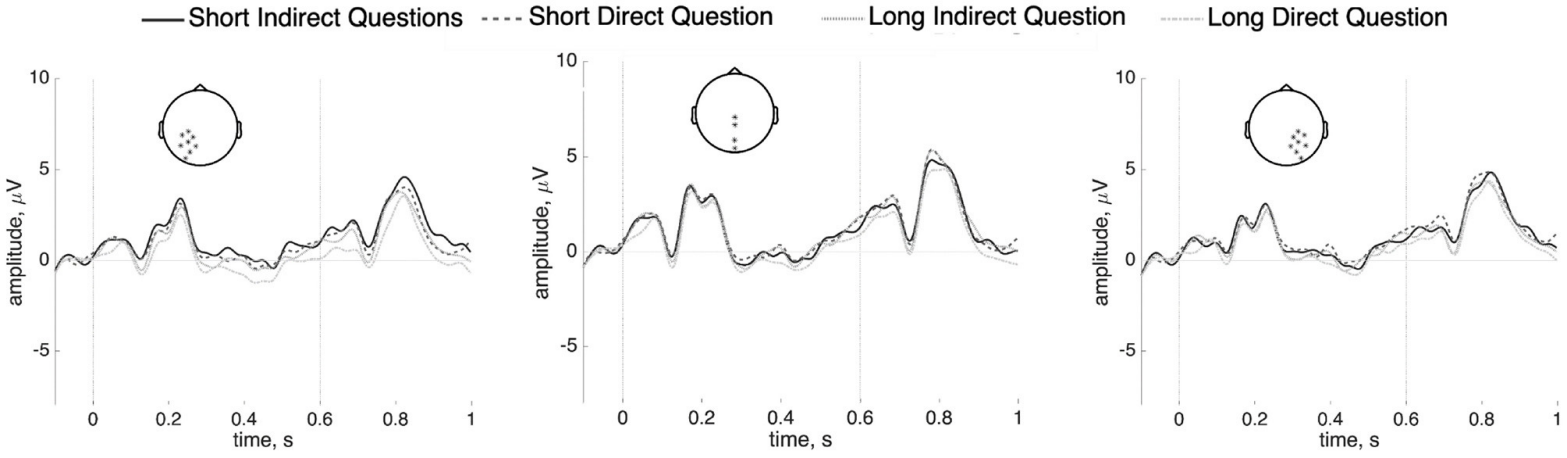
ERPs (*n* = 37) at the *wh*-word *naxie* “which” in the short/long indirect questions and the short/long direct questions at the posterior sites with a 100 ms prestimulus baseline.

Despite the absence of apparent anterior negativities or P600 effects relating to covert dependencies in Mandarin, we conducted a planned analysis separating out short from long dependencies at the target *wh*-word. A *t*-test was computed for the *wh*-word in the short and long conditions separately for indirect and direct questions. No effects were observed in either the short or long conditions (short: *p* = 0.69; long: *p* = 0.31). The Bayes factor analysis favors, albeit not strongly, the null models (short: 16.36; long: 2.39). This comparison is shown in Figure 9. The results of time-frequency analysis also did not reach statistical significance (*p* = 0.18). The figure is shown Supplementary Figure 8.

### 3.6. Assessing Effect Sizes of the SAN Through Simulation

As the previous results show, the SAN and the P600 appeared to be absent in Indirect questions, compared to Direct questions and the declarative counterparts. To test whether the absence of a significant effect may reflect an underpowered study, we conducted a *post-hoc* simulation test. In addition to serving our present goals, this approach may provide a way to assess replicability in ERP studies, an issue which has drawn increased attention recently though few studies examine the effect sizes that can be reliably detected with standard ERP analyses (see Cohen, 2017; Luck and Gaspelin, 2017; Thigpen et al., 2017; Boudewyn et al., 2018). Because ERPs comprise multiple parameters (e.g., amplitude over time, latency and duration for a given component, topographical distribution), there is not a simple test for whether an effect “replicates” across different studies. Furthermore, though most literature reports the *F*-value or *t*-value along with their *p*-value, Larson and Carbine (2017) show that only 40% of paper included effect sizes, 56% included mean values, and 47% included some estimate of variance. Rarely reporting such information impedes sample size calculation needed for conducting power analyses (Guo et al., 2014). Simulation-based approaches have been explored in prior work, but existing toolboxes, like BESA Simulator or Fieldtrip, simulate ERPs from scratch by making assumptions about the source model and noise model which may not reflect actual data.

Given these considerations, we test the sensitivity of our experiment using a novel simulation-based method which uses the observed single trial data to assess the statistical power across a range of plausible effect sizes and the number of participants. Using actual raw single trial data as the bases for the simulation allows for greater fidelity between simulated outcomes, and actual experimental outcomes. The procedure of the simulation is as follows:

1. The pre-processed single-trial data are randomly divided into three partitions that reflect conditions for each participant.
2. A stochastic effect *E* is drawn from a Gaussian distribution with parameters based on the component being evaluated and added to a subset of electrodes in one of the three partitions.
  - For the SAN, μ = {0.5, 1, 1.5, 2, 2.5, 3} and σ is derived from all trials at 250 ms in an anterior electrode per subject; *E* is added to the 24 anterior electrodes symmetrical around midline.
  - For the P600, μ = {0.5, 1, 1.5, 2} and σ is derived from all trials at 250 ms in an posterior electrode per subject; *E* is added to the 20 posterior electrodes that are symmetrical around midline.
3. Then the data are then averaged, and a group analysis is conducted as was for the experimental data.

The above steps (1–3) are repeated 100 times, yielding a distribution of statistical outcomes where the true effect *E* is known. Based on previous studies, the effect size for the SAN found in the *wh*-questions is approximately 1.5–3 mV (Fiebach et al., 2002; Phillips et al., 2005). Some studies testing for SAN effects did not report significant differences (McKinnon and Osterhout, 1996; Kaan et al., 2000), accordingly, we draw from a broader range of six different mean values (0.5, 1, 1.5, 2, 2.5, and 3) and three different numbers of participants (20, 30, and 37) were set to test the sensitivity of our methods to detect the SAN. As for the P600, the effect size is approximately 1.3–2 mV (Kaan et al., 2000; Phillips et al., 2005). Thus, four different mean values (0.5, 1, 1.5, and 2) and also three different numbers of participants (20, 30, and 37) were simulated.

**Figure 10:**
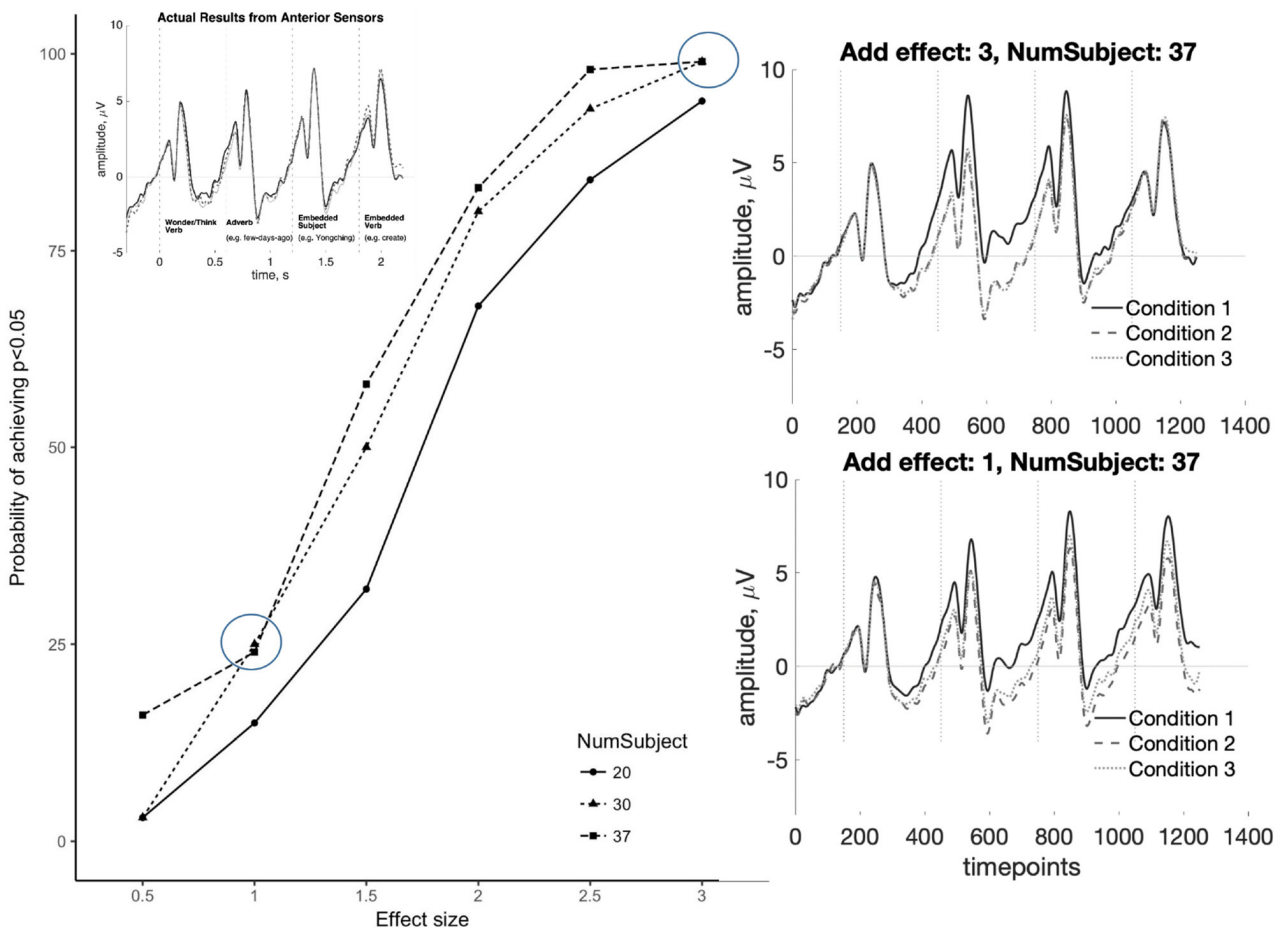
Simulation results for the SAN at six different effect sizes *E* (0.5, 1, 1.5, 2, 2.5, and 3 mV) and three different numbers of participants (20, 30, and 37). These show that SAN effects within the range of previously published results (≈ 1.5 mV) can be reliably detected in our dataset in >50% of runs. The observed null results (inset top-left) are visually distinct from effects as small as 1 mV (right panels).

**Figure 11:**
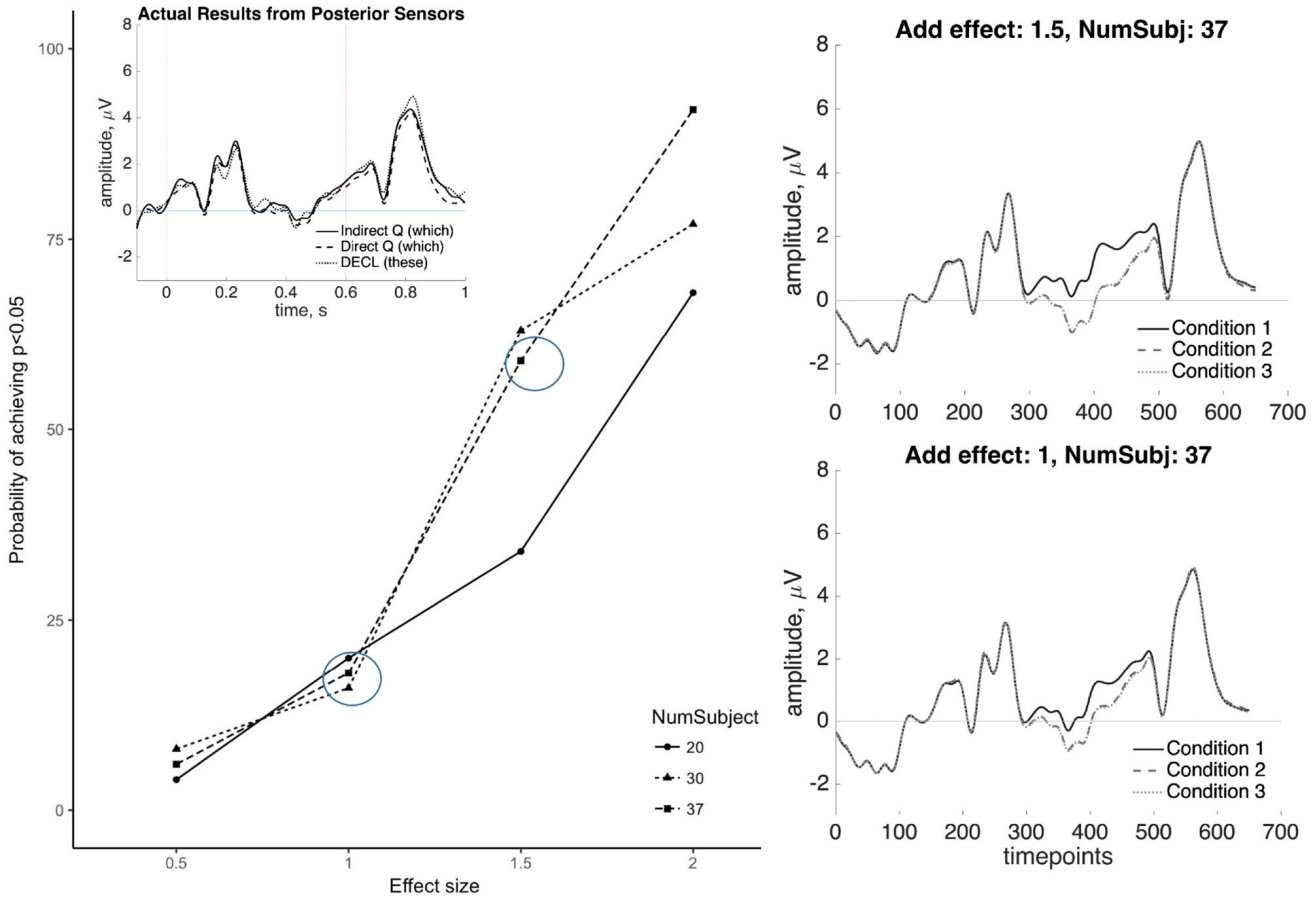
Simulation results for the P600 at four different effect sizes *E* (0.5, 1, 1.5, and 2 mV) and three different numbers of participants (20, 30, and 37). These show that P600 effects within the range of previously published results (≈ 1.5 mV) can be reliably detected in our dataset in >50% of runs. The observed null results (inset top-left) are visually distinct from effects as small as 1 mV (right panels).

The results of the simulations are shown in Figures 10, 11. For the SAN, results show that effects within the range of 0.5–3 mV, when added to random partitions of our actual data, can be detected using our statistical procedure with reasonably high reliability. For *E* = 2.5 mV, our procedure detects significance 90% of the time with *N* = 30 or *N* = 37. For *E* = 3.0 mV, significant effects are detected nearly 99% of the time with *N* = 30 or *N* = 37. Note that when *E* = 1.0 mV, a smaller effect than those reported in the SAN literature, significant effects are detected 25% of the time or less. As for the P600, for *E* = 2 mV, the simulation detects significance 90% of the time with *N* = 37. For *E* = 1.5 mV, effects can be detected nearly 60% of the time with *N* = 30 or *N* = 37. For *E* = 1 mV, a smaller effect than those reported in the P600 literature, significant effects are detected <25% of the time. The simulation results suggest that null statistical findings in the current study indicate a true effect of the sizes generally reported in the literature may indeed be absent in our data.

## 4. Discussion

In this study, we recorded EEG data during a word-by-word reading in Mandarin task to examine the functional role of the SAN and other ERP components that have been linked with dependency processing during language comprehension. In particular, by examining Mandarin covert *wh*-dependencies, we address the issue of whether the SAN reflects the maintenance of *wh*-features in working memory, or the maintenance of a contentful prediction. There are several key findings in this study. First, there was not strong evidence for a sustained negativity between the question-selecting verb and the *wh*-word in indirect questions. This result is inconsistent with the hypothesis that the SAN effect seen in prior studies reflects active maintenance of the *wh*-dependency itself. It is consistent with the hypothesis that memory resources used to store and retrieve the question-selecting feature cued by the main verb impose lower or no processing demands. Second, no P600 was found at the *wh*-word *naxie* “which,” relative to the demonstrative *zhexie* “these.” This result is most consistent with the hypothesis that the P600 is an indicator of the reactivation of the *wh*-filler: since the *wh*-word does not move overtly in Mandarin, such reactivation does not happen and thus there is no P600. Third, regarding the length effects, no P600 was elicited at the *wh*-word in the long condition, suggesting that the P600 reflects several sub-processes as a whole, not the length effect only. However, a sustained positivity was found in the long condition, and this unexpected result might, as we suggest below, indicate the inhibition of the expected incoming words.

Importantly, the principle results from the present study are based on the absence of the predicted ERP components. We contend that these null results indeed reflect some important aspects about how to interpret the functional role of the anterior negativity and the P600 in terms of the processing of the Mandarin *wh*-questions.

### 4.1. Consequences for the Functional Role of Anterior Negativities

As mentioned earlier, the anterior negativity correlates with the storage and the retention in working memory (Fiebach et al., 2002; Phillips et al., 2005). If the question-selecting verb serves as a cue and needs to be stored and retrieved in working memory after the *wh*-word is parsed, an anterior negativity is expected to be elicited in the indirect question. The results show that there is no SAN in the interval between the question-selecting verb and the *wh*-word, suggesting that the maintenance of the question feature of question-selecting verb in Mandarin *wh*-questions requires lower demands in working memory than the maintenance of a contentful *wh*-filler in English *wh*-questions. For the English *wh*-questions, the *wh*-word is encountered before the gap. Same as the Japanese *wh*-questions, the *wh*-word is also encountered before the question particle. Both English and Japanese *wh*-questions elicit an anterior negativity in the *wh*-dependencies. However, the *wh*-word is encountered after the question-selecting verb in Mandarin *wh*-question, suggesting different “memory loads” as a function of the different representations. That is, the memory load for the question-feature of the question-selecting verb is lower than the memory load for the *wh*-word in the English *wh*-questions.

Another possibility for the absence of the SAN might be due to the fact that the question-type verb can be followed by a non-*wh* NP to form a so-called “concealed question” (Nathan, 2006; Xiang et al., 2015). These are constituents that appear to be NPs following a question-selecting verb (e.g., She asked the price.). In such cases, the NP appears to denote a question (along the lines of what the price is). A Mandarin example is given in (8). However, it does not necessarily mean that the question-type verb does not need to form a dependency in Mandarin. In (8), the sentence semantically indicates “Zhangsan wonders what the price is.” That is, a *wh*-feature is still needed to be licensed covertly from a theoretical perspective. In addition, if the embedded clause in the indirect questions appears to be a non-interrogative clause, the whole sentence is considered to be ungrammatical, as shown in (9). Our experiment always included an embedded interrogative clause in Indirect questions and thus we reason that the parser is likely to form dependencies between the question-type verb and the *wh*-element.


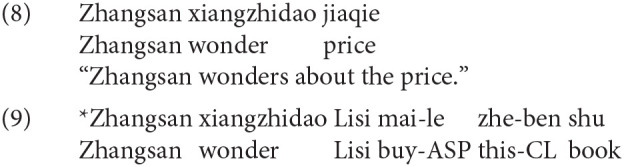


The above interpretation for the lack of anterior negativities in the Mandarin *wh*-questions carries two implications for how the parser treats cues for dependencies. First, if the anterior negativity does correlate with working memory demands, it is possible that language users do not need to store the question-selecting verb as a cue and then retrieve it. Instead of storing the cue for the wonder-type verb, under the expectation-based account, the parser uses cues from the main verb to make prediction for the upcoming words and build the expected syntactic structure. Xiang et al. (2015) also points out how the effect of differences in cue strength in wonder-type and think-type verbs for parsers remains unclear. Still, their study shows that the wonder-type verb can serve as a cue and supports the hypothesis that language users need to retrieve the cue for the wonder-type verbs and form the *wh*-dependencies in Mandarin. We suggest that our null results indicate that the SAN effect might correlate with the maintenance of contentful words with question feature, like *wh*-filler in English *wh*-questions, but not the maintenance or prediction of question dependency alone.

However, if we examine the Short and Long condition separately, a statistically reliable effect was found in the Long conditions. A sustained positivity was found in the Indirect question, compared to the Direct question. This result is in opposite direction of a SAN, and this unexpected finding may suggest that the question feature has an alternative effect on processing. Interestingly, previous studies have found the sustained anterior positivity correlates with the inhibition of highly expected words (DeLong et al., 2014). This may make sense for the Long indirect questions; once the parser has encountered the question-type verb, a *wh*-word is expected to appear soon. However, the fulfillment of this prediction is delayed in the Long condition due to the embedded clause between the main verb and the *wh*-word. Such delay might cause inhibition of the expectation for the target question feature, thus eliciting the sustained positivity. More fine-grained studies are needed for testing the interpretation of this sustained positivity. As one final caveat, our results cannot tease apart whether a parser needs to store the verb as a cue or only uses the verb to make prediction without storing it.

### 4.2. The Absence of the P600

Consistent with Ueno and Kluender's findings for Japanese *wh*-questions, we see no P600 effect at the *wh*-word in the Mandarin indirect questions. The Mandarin *wh*-questions does not have overt displacement for the *wh*-word and there is no need to refer back to the *wh*-filler. This result is consistent with the hypothesis that the P600 is an index for the reactivation of the *wh*-filler since there is no need to reactivate the *wh*-filler in the Mandarin *wh*-questions.

The absence of the P600 appears to be inconsistent with the hypothesis linking this component with thematic and syntactic integration between the main verb and the *wh*-filler under the assumption that *wh-*words impose stronger integration costs (Xiang et al., 2015). Indeed, this result seems at odds with Ueno and Kluender's view (2009) based on Japanese data that a covertly moved *wh*-element does not need to be integrated with the question particle in the processing of Japanese *wh*-questions. To reconcile these findings, we suggest, rather, that there is some integration between the question-selecting verb and the *wh*-element in the processing of the Mandarin *wh*-questions, but crucially thematic and/or syntactic integration that is indexed by the P600 is nearly equivalent when the parser encounters the *wh-* and non-*wh-* target words, leading to no P600 differences. On this particular formulation, the effects of retrieval interference for covert *wh-*questions identified by Xiang et al. (2015) may not modulate the P600 component.

As for the length effects, consistent with the results from Phillips et al. (2005), no reliable P600 effect was found at the *wh*-word in the long *wh*-dependency, compared to the short *wh*-dependency. This is consistent with the view from Phillips et al. (2005), suggesting that the P600 itself may not serve as an index of the length effect, but reflect several different processes simultaneously. Though the absence of both SAN and P600 in the current study, it is less clear to see whether the absence of the P600 is due to the insensitivity of the length effect or the similar integration difficulty in every conditions. Since the memory load resulting from the question feature carried by the question-type verb is not as heavy as the memory load resulting from the contentful *wh*-filler in the English *wh*-questions, the length effect might also be less significant.

### 4.3. Incidental Findings: P2

A significant early positivity was found at the *wh*-word, compared to the demonstrative *zhexie* “these.” A P2 is an earlier positive-going ERP component that distributed in the central-frontal regions at about 150–250 ms post-stimulus (Federmeier et al., 2005). A P2 can correlate with attention and the detection of visual stimuli and can relates to some levels of higher order processing (Luck and Hillyard, 1994; Federmeier et al., 2005). The amplitude of the P2 can be modulated by the sentence endings. If an expected word occurs in the sentence-final position, a larger P2 would be elicited than the one in the condition of an unexpected word in the sentence-final position. The P2 differences between the *wh*-word and the demonstrative might be a consequence of visual detection of lexical differences. The P2 effects found at the *wh*-word also suggest that participants did pay attention to the stimuli.

## 5. Conclusion

In contrast to prior work, no sustained anterior negativity was found between the main verb and the *wh*-word, nor was SAN or P600 observed at the *in-situ wh*-word. The absence of sustained negativities in Mandarin *wh*-questions suggests two possibilities. The first is that there is no need to store the question-selecting verb as a cue and then retrieve it. The second is that there are lower working memory demands for the storage and retrieval of the question feature of the question-selecting verb, which is different from the storage of the contentful *wh*-word in the processing of English and Japanese *wh*-questions. We did observe a sustained positivity for the longer *wh-*dependencies, which could suggest that a prediction of some sort is being maintained and causing to lexical inhibition when that prediction is not rapidly satisfied. Taken together, these results narrow down the kind of memory mechanisms indexed by ERP components, like the SAN, during language comprehension.

## Data Availability Statement

The raw data supporting the conclusions of this article will be made available by the authors, without undue reservation.

## Ethics Statement

The studies involving human participants were reviewed and approved by University of Michigan Health Sciences and Behavioral Sciences Institutional Review Board. The patients/participants provided their written informed consent to participate in this study.

## Author Contributions

C-WL: conceptualization, material design, acquisition of data, data analysis, data curation, interpretation of data, manuscript preparation, and visualization. JB: conceptualization, interpretation of data, manuscript review and editing, funding acquisition, and project administration. All authors contributed to manuscript revision, read and approved the submitted version.

## Conflict of Interest

The authors declare that the research was conducted in the absence of any commercial or financial relationships that could be construed as a potential conflict of interest.
