# Supplementary material for: EEG Correlates of Long-Distance Dependency Formation in Mandarin Wh-Questions
Source: Front Hum Neurosci. 2021 Feb 5;15:591613. doi: 10.3389/fnhum.2021.591613 (PMC7892779; doi:10.3389/fnhum.2021.591613)
Supplement: Supplementary file 1 [file Data_Sheet_1.PDF]

Supplementary Material

1 TIME-FREQUENCY ANALYSIS

For the interval analysis, epochs spanned the 2,200 ms between the onset of the main verb and the onset of the *wh*-word along with a 300 ms pre-stimulus baseline. For the target-word analyses, 1,000 ms epochs were time-locked to the onset of the *wh*-word or demonstrative with a 300 ms pre-stimulus baseline. As for the length effects, each trial was time-locked to the onset of the target word in a 3,600 ms time-window with a 300 ms pre-stimulus baseline. In order to avoid boundary effects, an additional 100 ms of data was added to the beginning and end of the epochs. In addition, each epoch was padded a 2,000-ms zero on both sides. Morlet wavelets with seven cycles were applied (Lachaux et al., 1999) to identify changes in spectral power over time. Power values were computed from 2 Hz to 50 Hz with a 0.5-Hz step and from the interest of the time-window with a 50-ms step. For statistical analysis, cluster-based permutation tests were conducted by applying Monte-Carlo simulation in 1,000 times to the theta and alpha bands. Tests with $p < 0.05$ were clustered and a minimum of three neighboring electrodes were considered as a cluster.

1.1 Results

For the interval analysis, Indirect Questions, Direct Questions, and Declaratives were compared across the interval between the main verb and the *wh*-word. The results of time-frequency analysis also did not reach significance ($p = 0.87$). The figure is shown in S1. Each word in the interval (i.e. Adverb, Embedded subject, and Embedded verb) was also compared. The results of time-frequency analysis also did not reach significance for each word (Adverb: $p = 0.39$; Embedded subject: $p = 0.78$; Embedded verb: $p = 0.82$). The averaged time-frequency results at anterior regions are shown for the adverb (S2), the embedded subject (S3), and the embedded verb (S4). As for the *wh*-word vs. demonstrative, we also did not find statistical significance ($p = 0.44$). S5 shows the averaged time-frequency results from the posterior regions. For the length effects, Indirect questions and Direct questions were compared in both short and long conditions. The results of time-frequency analysis show no statistical differences in both short ($p = 0.72$) and long comparisons ($p = 0.59$). The figures are shown in S6 for the short condition and S7 for the long condition. We also compared the *wh*-word in Indirect questions and Direct questions for the length effects separately. Results also did not reach statistical significance ($p = 0.18$). S8 shows the averaged results from the posterior regions.

Overall, we don't see any statistically reliable effects in the theta and alpha range that specifically relate to memory mechanisms. The direct questions seem to have greater power in higher frequencies, although these are not statistically reliable (Figure S5 and S8).

FIGURES

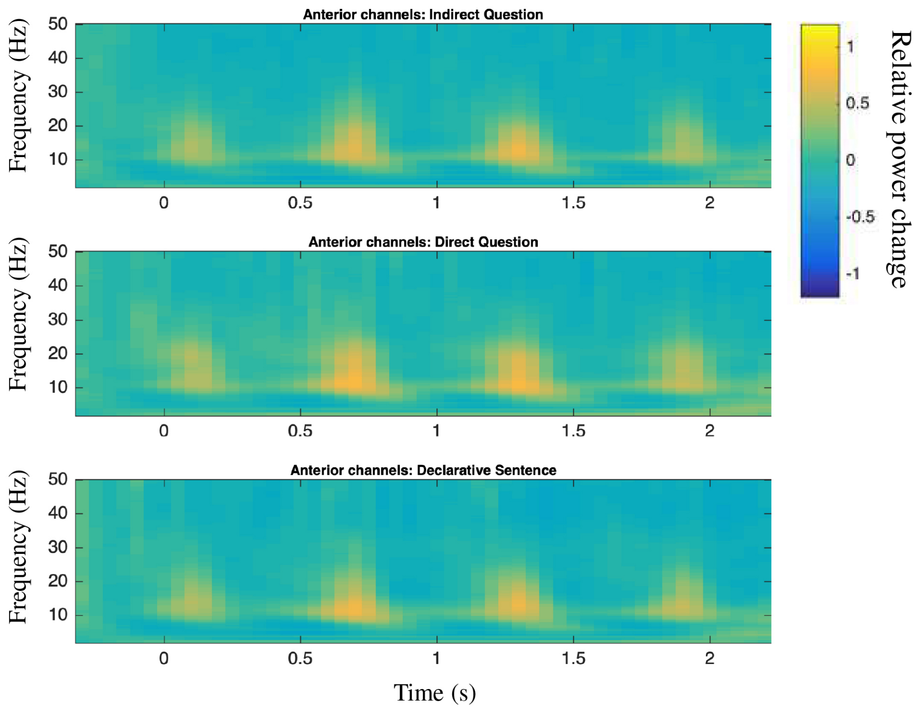

Figure S1. The interval between the main verb and the *wh*-word/demonstrative in Indirect Questions, Direct questions, and Declarative Sentences at anterior sensors (300 ms baseline computed at the beginning of the interval). There are no statistically reliable effects.

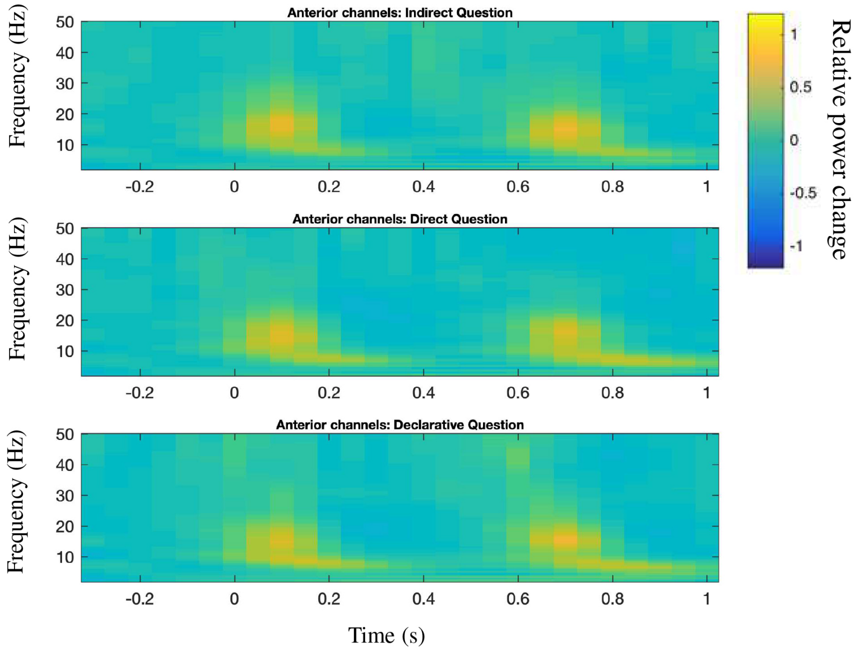

Figure S2. The averaged time-frequency results for the adverb in the anterior regions. There are no statistically reliable effects.

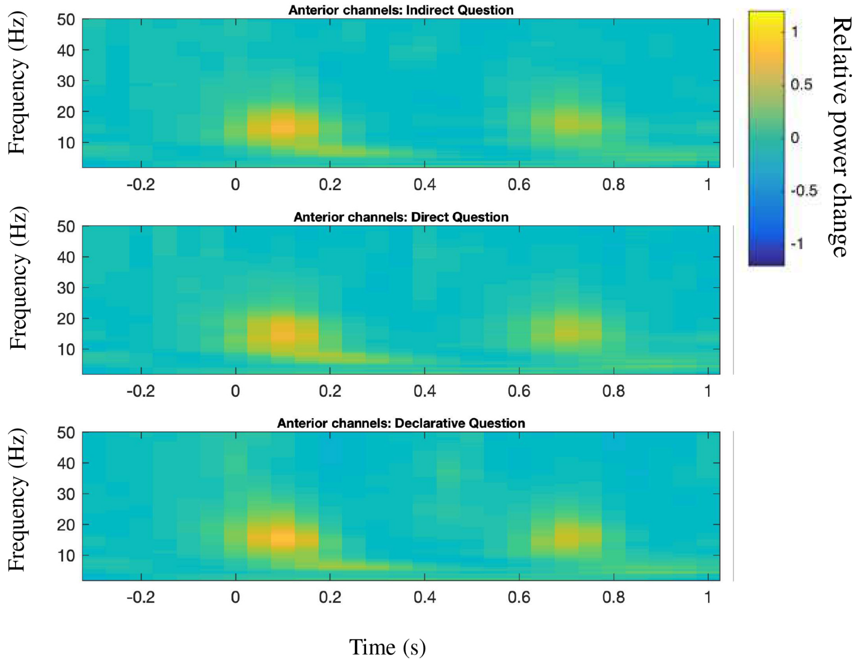

Figure S3. The averaged time-frequency results for the embedded subject in the anterior regions. There are no statistically reliable effects.

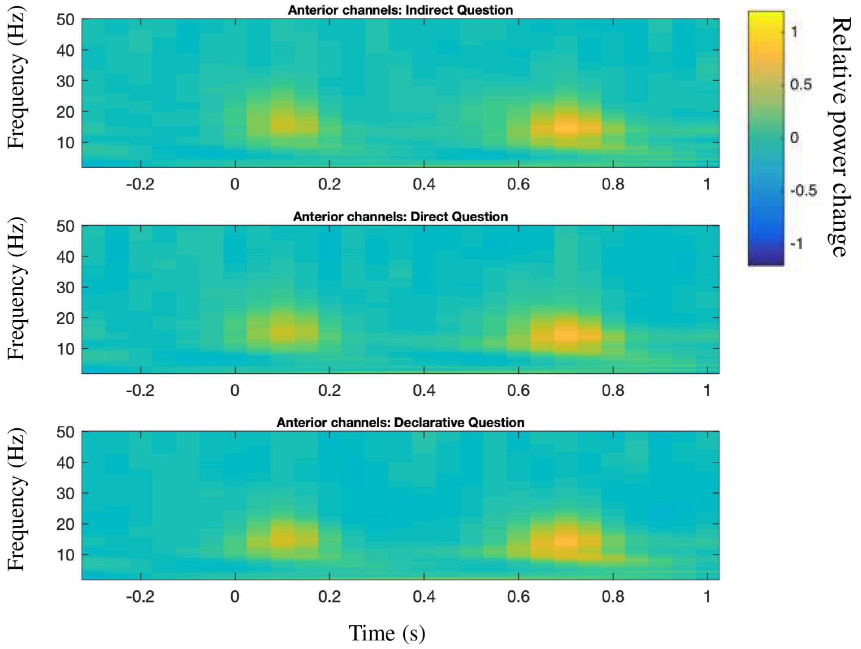

Figure S4. The averaged time-frequency results for the embedded verb in the anterior regions. There are no statistically reliable effects.

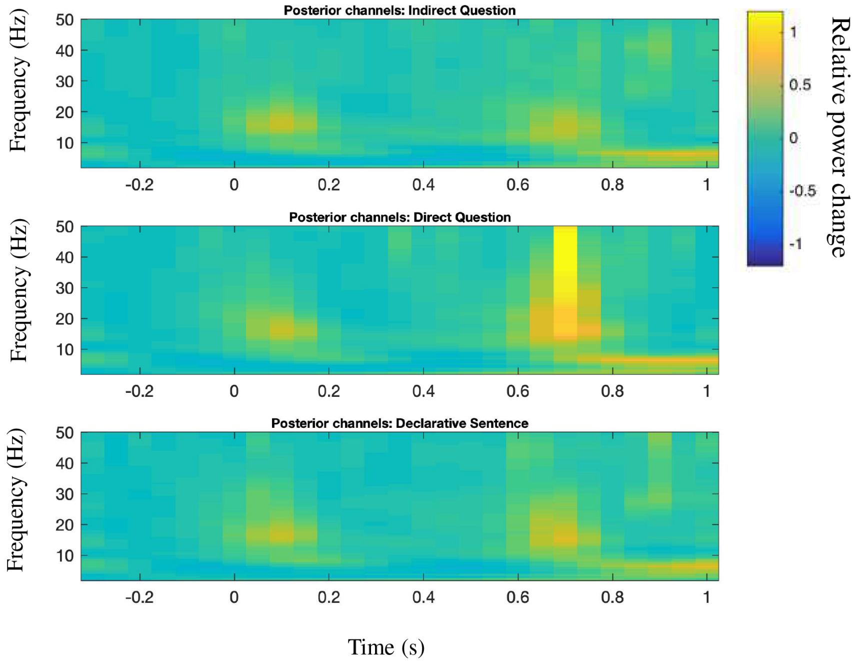

Figure S5. The averaged time-frequency results for the *wh*-word *naxie* "which" in the indirect questions/direct questions and the demonstrative *zhexie* "these" in the declarative sentences. There are no statistically reliable effects.

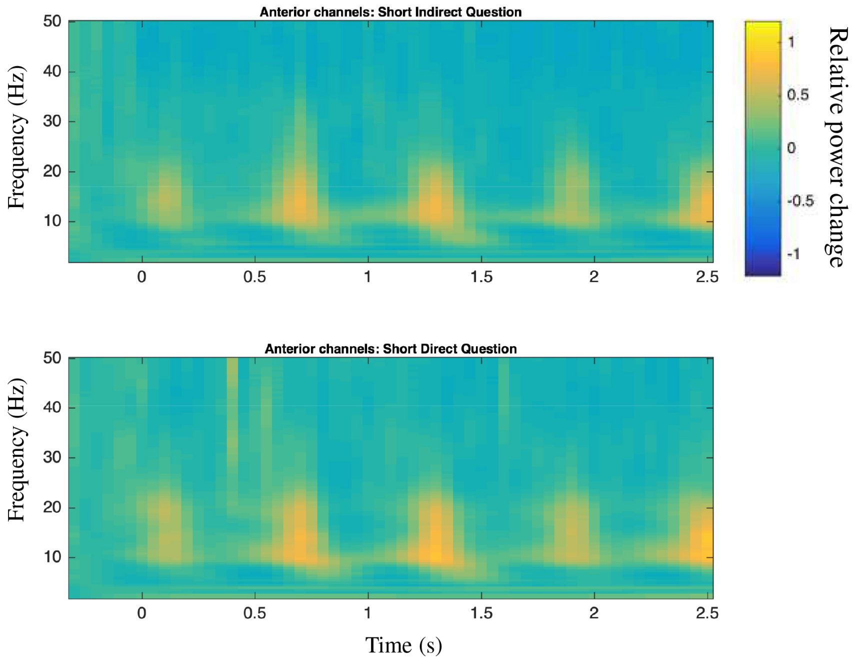

Figure S6. The averaged time-frequency results for the interval between the main verb and the *wh*-word *naxie* "which" in the indirect questions and the direct questions for the short condition. There are no statistically reliable effects.

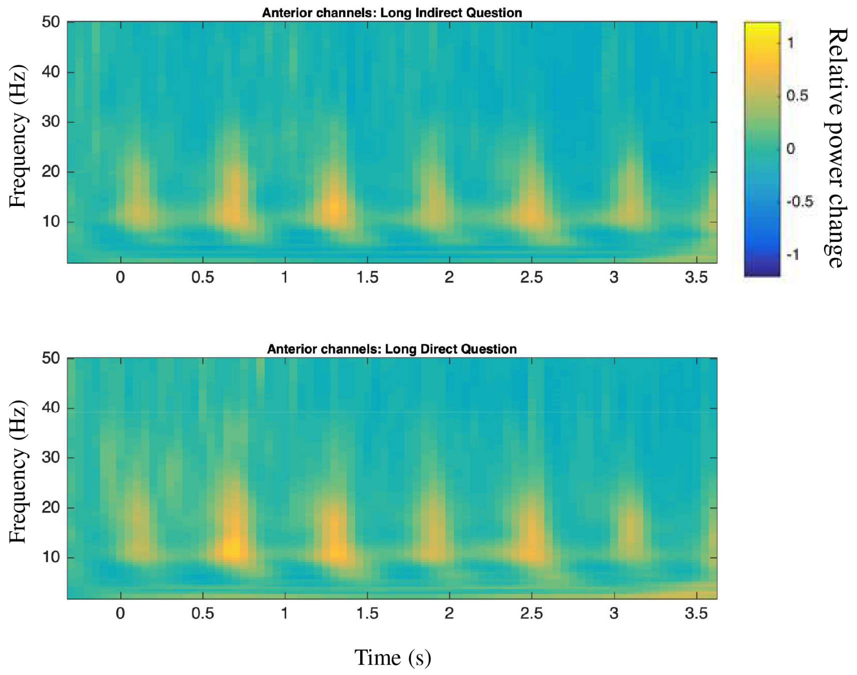

Figure S7. The averaged time-frequency results for the interval between the main verb and the *wh*-word *naxie* "which" in the indirect questions and the direct questions for the long condition. There are no statistically reliable effects.

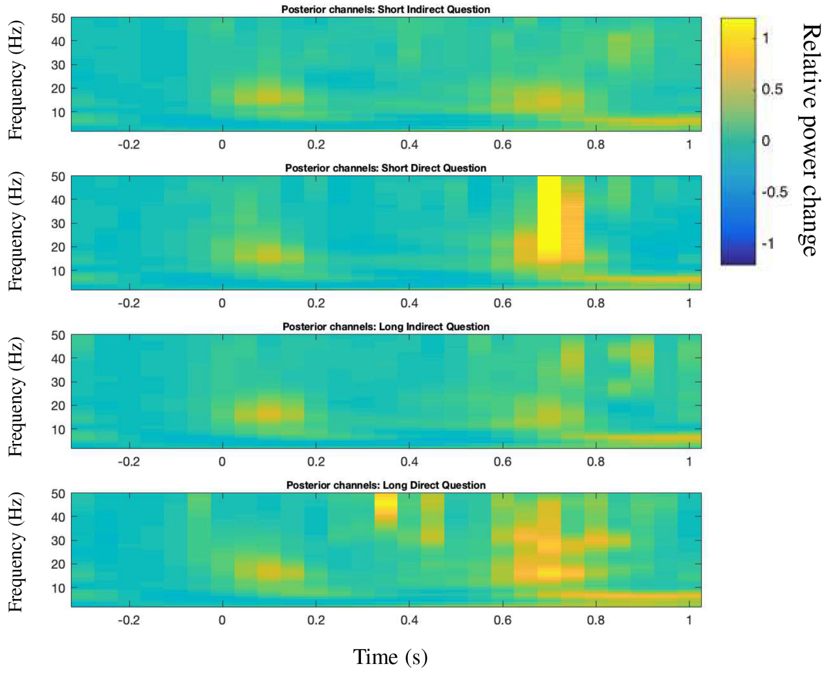

Figure S8. The averaged time-frequency results at the *wh*-word *naxie* "which" in the short/long indirect questions and the short/long direct questions at the posterior sites. There are no statistically reliable effects.

Verb	Gloss	Type	Stroke (First)	Stroke (Second)	Stroke (sum)	1st Char. Log freq.	2nd Char. Log freq.	Word freq. (log)
質問	To question	[+wh]	15	11	26	3.77	4.81	1.99
追問	To question closely	[+wh]	10	11	21	3.9	4.81	1.77
逼問	To question intensely	[+wh]	13	11	24	3.49	4.81	0
盤問	To interrogate	[+wh]	15	11	26	3.63	4.81	2.31
詢問	To inquire	[+wh]	13	11	24	3.16	4.81	2.78
查問	To inquire	[+wh]	9	11	20	4.49	4.81	1.4
反問	To ask in reply	[+wh]	4	11	15	4.2	4.81	1.49
試問	To try to ask	[+wh]	13	11	24	4.43	4.81	0.78
責問	To blame and ask	[+wh]	11	11	22	4	4.81	0.9
拷問	To question via torture	[+wh]	9	11	20	2.71	4.81	2.01
審問	To interrogate	[+wh]	15	11	26	3.8	4.81	2.62
提到	To mention	[-wh]	12	8	20	4.38	5.46	3.12
建議	To suggest	[-wh]	9	20	29	3.99	4.17	3.1
强調	To emphasize	[-wh]	11	15	26	4.2	4.16	2.4
以爲	To think	[-wh]	5	9	14	5.27	5.4	4.22
説出	To say	[-wh]	14	5	19	5.45	5.11	0
公布	To announce	[-wh]	4	5	9	4.59	4.26	2.66
認爲	To think	[-wh]	14	9	23	4.73	5.4	4.4
報導	To report	[-wh]	12	16	28	4.34	3.86	2.53
談到	To mention	[-wh]	15	8	23	4.55	5.46	1.32
看到	To see	[-wh]	9	8	17	5.33	5.46	4.43
發現	To find	[-wh]	12	11	23	4.94	5.14	4.29
揭曉	To reveal	[-wh]	12	16	28	3.23	3.28	2.86
觀察	To observe	[-wh]	25	14	39	3.95	4.22	3.04
澄清	To clarify	[-wh]	15	11	26	2.39	4.34	2.35
聽到	To listen	[-wh]	22	8	30	4.91	5.46	3.83
推斷	To infer	[-wh]	11	18	29	3.82	3.96	2.18
指出	To point out	[-wh]	9	5	14	4.34	5.11	2.62
形容	To describe	[-wh]	7	10	17	3.84	4.05	2.8
宣布	To announce	[-wh]	9	7	16	3.59	4.26	3.18
證實	To confirm	[-wh]	19	14	33	4.48	4.76	3
調查	To investigate	[-wh]	15	9	24	4.16	4.49	3.62

APPENDIX 1. MAIN VERBS IN THE STIMULI

Verb selection criteria	**Question-selecting verb**	**Non-question verb**
**Number of strokes** ($t(29.8) = 0.34$, $p > .05$)	M = 22.55, SD= 3.39	M = 23.19, SD = 7.24
**Log word frequency** ($t(21.7) = 4.59$, $p < .001$)	M = 1.8, SD= 0.67	M = 3.1, SD = 0.82
**Log frequency of the first character** ($t(25.8) = 2.02$, $p > .05$)	M = 3.78, SD = 0.53	M = 4.27, SD = 0.77
**Log frequency of the second character** ($t(20) = 1.04$, $p > .05$)	M = 4.81, SD = 0	M = 4.66, SD = 0.67

APPENDIX 2. VERB SELECTION CRITERIA

REFERENCES

Lachaux, J., Rodriguez, E., Martinerie, J., and Varela, F. J. (1999). Measuring phase synchrony in brain signals. *Human Brain Mapping* 8, 194–208. doi:https://doi.org/10.1002/(SICI)1097-0193(1999)8:4<194::AID-HBM4>3.0.CO;2-C
